# OGS-MA114 ameliorates FUS mutant-associated ALS pathology by activating PINK1-dependent mitophagy

**DOI:** 10.1080/19768354.2026.2736980

**Published:** 2026-11-10

**Authors:** Seong Hyun Kim, Jun Hee So, Yunkyoung Lee, Na Yeon Park, Jung Sang Park, Pansoo Kim, Ji-Eun Bae, Doo Sin Jo, Hae-Chul Park, Dong-Hyung Cho

**Affiliations:** a School of Life Sciences, BK21 FOUR KNU Creative BioResearch Group, Kyungpook National University, Daegu, Republic of Korea; b Zebrafish Translational Medical Research Center, Department of Biomedical Sciences, Korea University, Ansan, Republic of Korea; c Organelle Institute, Kyungpook National University, Daegu, Republic of Korea; d ORGASIS Corp., Suwon, Republic of Korea

**Keywords:** OGS-MA114, mitophagy, FUS^P525L^, amyotrophic lateral sclerosis, neuroprotection

## Abstract

Mitochondrial dysfunction and impaired mitophagy are increasingly recognized as central contributors to pathogenesis of amyotrophic lateral sclerosis (ALS). In this study, we identified OGS-MA114 (C₂₃H₁₉ClN₄O₃S), as a compound that activates mitophagy through a bioactive chemical library screen in SH-SY5Y cells. OGS-MA114 significantly enhanced autophagic and mitophagic flux, increased Ser65-phosphorylated ubiquitin levels, and promoted Parkin recruitment to damaged mitochondria, indicating activation of the PINK1/Parkin pathway. In SH-SY5Y cells expressing the ALS-associated FUS^P525L^ mutant, OGS-MA114 restored mitochondrial membrane potential, reduced cleaved caspase-3 levels, and improved cell viability under mitochondrial stress conditions. Furthermore, in a transgenic zebrafish model expressing mutant FUS^R521C^, OGS-MA114 significantly improved locomotor activity and survival while reducing cell death in the spinal cord. These findings suggest that OGS-MA114 ameliorates ALS-associated pathological phenotypes by enhancing PINK1-dependent mitophagy. Thus, pharmacological modulation of mitophagy may provide a useful basis for further investigation of ALS and -related neurodegenerative disorders.

## GRAPHICAL ABSTRACT

**Figure:**
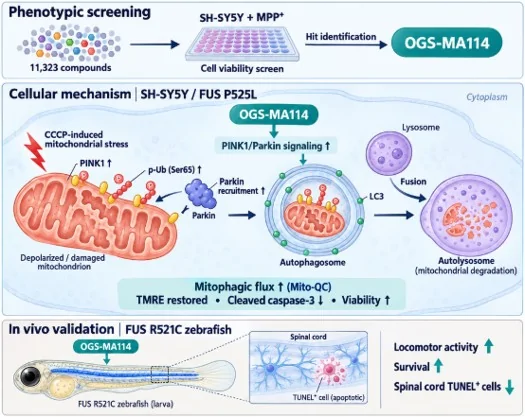

## Introduction

Amyotrophic lateral sclerosis (ALS) is a progressive and fatal neurodegenerative disorder characterized by the selective loss of upper and lower motor neurons, leading to muscle weakness, paralysis, and ultimately respiratory failure. Currently approved therapies provide only modest clinical benefit (Mead et al. 2023). Mitochondrial dysfunction is increasingly recognized as a central feature of ALS pathology, contributing to oxidative stress, bioenergetic failure, axonal vulnerability, and motor neuron degeneration (Li et al. 2025; Li et al. 2026; Madruga et al. 2021). Because motor neurons are highly dependent on mitochondrial integrity to sustain long axons and persistent energetic demands, disruption of mitochondrial quality control has particularly detrimental consequences in ALS (Pickles et al. 2018; Xie et al. 2025).

Among ALS-associated genes, fused in sarcoma (*FUS*) is of particular interest because pathogenic mutations disrupt its normal nuclear localization and promote aberrant cytoplasmic accumulation (Vance et al. 2009; Dormann et al. 2010). Mutant FUS undergoes cytoplasmic mislocalization, which not only impairs its normal functions in RNA metabolism but also promotes the formation of persistent stress granules (SGs) associated with neuronal toxicity (Deng et al. 2010; Park and Kim 2025; Kim et al. 2026). In addition to the highly aggressive FUS^P525L^ mutation, ALS-associated FUS^R521^ variants, including FUS^R521C^ and FUS^R521H^, have been reported to disrupt nucleocytoplasmic localization and SG dynamics (Deng et al. 2010; Dormann et al. 2010).

Mitophagy, the selective autophagic elimination of damaged or unnecessary mitochondria, is an essential mitochondrial quality control system that maintains cellular homeostasis under various pathological conditions (Youle and Narendra 2011; Pickles et al. 2018). Among the several mitophagy pathways, PINK1/Parkin-mediated mitophagy is a critical mitochondrial quality-control mechanism. The PINK1/Parkin pathway is initiated by the stabilization of PINK1 on the outer mitochondrial membrane (OMM) following mitochondrial damage. Accumulated PINK1 phosphorylates ubiquitin and Parkin, promoting the recruitment of cytosolic Parkin to damaged mitochondria. Activated Parkin then conjugates ubiquitin chains to OMM proteins, marking damaged mitochondria for elimination, and recruitment of autophagy receptors that facilitate lysosomal degradation (Youle and Narendra 2011; Kane et al. 2014; Lazarou et al. 2015). Autophagy receptors such as optineurin (OPTN) are integral to this pathway, and ALS-associated defects in OPTN-dependent mitochondrial clearance further support the mechanistic relevance of impaired PINK1-Parkin signaling to neurodegeneration (Wong and Holzbaur 2014). In neurons, disruption of this mitophagic pathway may exacerbate oxidative damage and cell death (Pickles et al. 2018; Madruga et al. 2021; Park and Kim 2025).

Recent studies have shown that PINK1 and Parkin modulate FUS mutant-induced neurodegeneration, indicating that mitochondrial quality control is a downstream consequence of proteotoxic stress as well as an active determinant of disease severity (Chen et al. 2016). Notably, natural and bioactive compounds, including urolithin A, spermidine, and several herbal terpenoids, have emerged as potential modulators of autophagy and mitophagy in neurodegenerative diseases and aging-related conditions (Ahsan et al. 2021; Civiletto et al. 2025; Kim et al. 2025; Borsky et al. 2026). For example, a recent cross-species study of ALS identified isoginkgetin as a robust mitophagy enhancer that promotes PINK1/Parkin-dependent mitochondrial clearance and ameliorates ALS phenotypes in animal models and patient iPSC-derived motor neurons, supporting phenotypic screening for mitophagy activation as an effective drug discovery strategy (Li et al. 2025; Li et al. 2026). In parallel, emerging evidence suggests that PINK1 may act more broadly as a regulator of organelle quality control beyond canonical mitophagy, further supporting the therapeutic potential of PINK1-centered stress-response pathways (Jo et al. 2026).

Therefore, we performed a compound library screen to identify molecules that suppress cell death with mitophagy activation. From the screening, we identified OGS-MA114 (C₂₃H₁₉ClN₄O₃S), as a novel mitophagy-activating compound and investigated whether OGS-MA114 enhances PINK1-dependent mitophagy. In addition, OGS-MA114 treatment reduced oxidative stress-associated toxicity and ameliorated FUS-mutant ALS phenotypes in cellular and zebrafish models.

## Materials and methods

### Reagents

MPP^+^ iodide (MPP^+^, D048), Bafilomycin A1 (Baf A1; B1793), and CCCP (C2759) were purchased from Sigma-Aldrich (St. Louis, MO, USA). Tetramethylrhodamine ethyl ester perchlorate (TMRE, T669) was purchased from Invitrogen (Carlsbad, CA, USA). OGS-MA114 [N-(3-acetylphenyl)-2-(1-(4-(4-chlorophenyl)thiazol-2-yl)−5-hydroxy-3-methyl-1H-pyrazol-4-yl)acetamide] was synthesized by BioDuro (Jiangsu, China) company in a five-step process and used for necessary biological assays or evaluations. LC/MS: 467.1 [M + H]^+^. ^1^H NMR (400 MHz, DMSO) δ 12.22 (s, 1H), 10.30 (s, 1H), 8.21 (s, 1H), 8.03 (d, J = 8.4 Hz, 2H), 7.87-7.82 (m, 2H), 7.66 (d, J = 7.6 Hz, 1H), 7.54-7.44 (m, 3H), 3.38 (s, 2H), 2.55 (s, 3H), 2.28 (s, 3H).

### High-throughput cell viability screening

A total of 11,323 compounds (ChemDiv, San Diego, CA, USA) from four chemical libraries were screened including antiviral compounds (*n* = 3,200), autophagy-related compounds (*n* = 3,200), kinase regulators (*n* = 856), and standard compounds (*n* = 4,067). SH-SY5Y cells were seeded in 384-well plates and co-treated with 2 mM MPP^+^ and each test compound at 10 μM for 24 h. Cell viability was assessed using the CellTiter 96® AQueous One Solution Cell Proliferation Assay (Promega, Madison, WI, USA). MTS reagent was added directly to each well, and the plates were incubated at 37 °C in 5% carbon dioxide (CO₂) until the absorbance difference between the negative (Nec, cell-free blank) and positive (Poc, untreated cells) controls reached 1.0. Absorbance was then measured at 490 nm using a microplate reader. Viability (%) for each plate was normalized to the on-plate positive (100%) and negative (0%) controls. The primary hit cutoff was defined as the mean viability of MPP^+^-only wells plus 3 SD; compounds yielding ≥ 75% viability were classified as strong hits. Assay quality was monitored using the Z′-factor calculated for each plate.

### Cell culture and establishment of stable cell lines

SH-SY5Y neuroblastoma cells were obtained from ATCC (Manassas, VA, USA). The EGFP-Parkin plasmid was kindly provided by Dr. Noriyuki Matsuda (Tokyo Metropolitan Institute of Medical Science, Japan). The ptf-Galectin3 and mCherry-Fis1 expression plasmids were purchased from Addgene (plasmid no. 64149 and 182580; deposited by Dr. Tamotsu Yoshimori, Osaka University, Japan, and Dr. Gia Voeltz, University of Colorado, Denver, CO, USA, respectively). The mRFP1-EGFP-Fis1(101–152) (Mito-QC) reporter construct was generated by replacing the Galectin3 coding sequence in ptf-Galectin3 with the Fis1(101–152) fragment, which was PCR-amplified from mCherry-Fis1 and inserted in-frame downstream of the mRFP1-EGFP tandem fluorescent tag. The resulting construct was verified by restriction digestion and DNA sequencing.

To generate mitophagy reporter cell lines stably expressing mRFP1-EGFP-Fis1(101–152) (SH-SY5Y/Mito-QC) or EGFP-Parkin (SH-SY5Y/EGFP-Parkin), SH-SY5Y cells were transfected with the corresponding plasmids using Lipofectamine 2000 (Invitrogen) according to the manufacturer’s protocol. Stable transfectants were selected with G418 (1.25 mg/mL) for 7 days, and single-cell clones were isolated and screened by fluorescence microscopy (IX71, Olympus, Tokyo, Japan) to confirm stable expression. The EGFP-FUS^P525L^ and EGFP-FUS^WT^ plasmids were generously provided by Dr. Jin-A Lee (Hannam University, Korea). Stable SH-SY5Y cell lines expressing EGFP-FUS^WT^ or EGFP-FUS^P525L^ were generated by lentiviral transduction in Dr. Komatsu’s laboratory (Juntendo University, Japan). Briefly, lentiviral particles were produced in HEK293 T cells by transfection with the pEGFP-FUS^WT^ or pEGFP-FUS^P525L^ lentiviral vector using Lipofectamine LTX (Invitrogen). Virus-containing medium was collected and transferred to SH-SY5Y cells for transduction. Transduced cells were selected with puromycin (1.5 µg/mL) for 3 days. Cells were cultured at 37 °C in a 5% CO_2_ incubator and maintained in Dulbecco’s Modified Eagle’s Medium (DMEM; LM 001-05, WELGENE, Gyeongsan, Korea) supplemented with 10% fetal bovine serum (16000-044, Gibco, Grand Island, NY, USA) and 1% penicillin–streptomycin (LS 202-02, WELGENE).

### Western blotting and ELISA

For immunoblotting, cells were lysed in lysis buffer and mixed with 2X Laemmli sample buffer (Bio-Rad, Hercules, CA, USA). Protein concentrations were determined using the Bradford assay (Bio-Rad) according to the manufacturer’s instruction. Then, the samples were separated by SDS-PAGE and transferred onto PVDF membranes (Bio-Rad). Membranes were blocked with 4% skim milk (MBcell, Seoul, Korea) in TBST [25 mM Tris-base (GenDEPOT, Katy, TX, USA), 140 mM NaCl (GenDEPOT), and 0.05% Tween® 20 (Sigma)] and then incubated with the indicated primary antibodies: anti-caspase-3 (1:3,000, #9668, Cell Signaling Technology, Danvers, MA, USA), anti-cleaved caspase-3 (1:3,000, #9661, Cell Signaling Technology), anti-TOM20 (1:2,000; sc-17764, Santa Cruz Biotechnology, Dallas, TX, USA), anti-ubiquitin (1:2,000; sc-8017, Santa Cruz Biotechnology), anti-phospho-ubiquitin (phospho-Ser65) (1:1,000; ab309155, 30H3/30K1, Abcam, Cambridge, UK), anti-LC3B (1:5,000, NB100-2220, Novus Biologicals, Littleton, CO, USA), anti-PINK1 (1:1,000; BC100-494, Novus Biologicals), anti-ACTB/β-actin (1:10,000, 66009-1-Ig, Proteintech, Rosemont, IL, USA). For protein detection, the membranes were incubated with HRP-conjugated secondary antibodies (1:5,000; #7076S and #7074S, Cell Signaling Technology). Signals were visualized using Luminograph I (ATTO, Tokyo, Japan).

For the ELISA, cells were washed with Dulbecco’s phosphate-buffered saline (DPBS; LB 001–02, WELGENE) and lysed directly in each well using lysis buffer (#9803, Cell Signaling Technology) supplemented with a protease inhibitor cocktail. Cell lysates were collected by scraping and clarified by centrifugation. Protein concentrations were determined using the Bradford assay (Bio-Rad) according to the manufacturer’s instruction. Phospho-ubiquitin (Ser65) levels in the cell lysates were determined using PathScan® Phospho-Ubiquitin (Ser65) Sandwich ELISA Kit (#28384, Cell Signaling Technology) according to the manufacturer’s instructions. Absorbance was measured at 450 nm using a Synergy H1 microplate reader (BioTek, Santa Clara, CA, USA).

### TMRE assay and cell viability assay

Changes in mitochondrial membrane potential were evaluated using tetramethylrhodamine ethyl ester (TMRE; T669, Invitrogen), a fluorescent probe that accumulates in mitochondria in a membrane potential-dependent manner. SH-SY5Y, SH-SY5Y/GFP-FUS^WT^, and SH-SY5Y/GFP-FUS^P525L^ cells were seeded in 96-well clear-bottom black-walled plates. The cells were then treated with OGS-MA114 (20 μM) in the presence or absence of CCCP (5 μM) for 24 h. After treatment, the cells were incubated with TMRE (200 nM) diluted in complete culture medium at 37 °C for 30 min in the dark. The cells were then washed twice with DPBS (LB 001–02, WELGENE) to remove excess dye, and TMRE fluorescence intensity was measured using a Synergy H1 microplate reader (BioTek) at excitation and emission wavelengths of 550 and 580 nm, respectively. A decrease in TMRE fluorescence intensity was interpreted as indicative of mitochondrial membrane depolarization, and impaired mitochondrial function.

Cell viability was measured using a Cell Counting Kit-8 (CCK-8, Dojindo, Kumamoto, Japan). SH-SY5Y, SH-SY5Y/GFP-FUS^WT^, and SH-SY5Y/GFP-FUS^P525L^ cells were seeded in 96-well plates and incubated at 37 °C for 48 h. The cells were then treated with MPP^+^ (2 mM) or CCCP (5 μM), with or without OGS-MA 114 (20 μM), for 24 h. CCK-8 solution (10 μL per well) was subsequently added, and the cells were incubated for an additional 1 h. Absorbance was measured at 450 nm using a Synergy H1 microplate reader (BioTek).

### Zebrafish maintenance and generation of transgenic zebrafish

All animal experimental procedures were approved by the Korea University Institutional Animal Care and Use Committee (KOREA-2023-0176-C1) and conducted in accordance with the animal experimentation guidelines of the Korea National Veterinary Research and Quarantine Service. Zebrafish were maintained at 28.5 °C under a 14-h light/10-h dark cycle. Embryos and larvae were raised in egg water or embryo medium (EM), and developmental stages were defined as days post-fertilization (dpf). To establish neuron-specific expression of ALS-associated mutant *FUS*, we generated the driver line *Tg(HuC:QFDBD-2xAD-VP16)*, in which the QFDBD transactivator is driven by the neuron-specific HuC promoter (Park et al. 2000). This driver line was crossed with the effector line *Tg* (*13XQUAS:ZsGreen-p2a-hFUS^R521C^*), which enables expression of human mutant FUS^R521C^ together with ZsGreen. Double-transgenic zebrafish larvae expressing hFUS^R521C^ in neurons are hereafter referred to as *HuC*:mtFUS.

OGS-MA114 was dissolved in DMSO to prepare a 10 mM stock solution, and diluted in EM to a final working concentration of 5 μM. *HuC*:mtFUS embryos were treated with 5 μM OGS-MA114 from 1 to 5dpf. Vehicle-treated *HuC*:mtFUS embryos exposed to 0.05% DMSO served as disease controls. Sibling embryos were maintained under the same experimental conditions and used as control animals.

### Locomotor behavior analysis and survival analysis in zebrafish

Locomotor behavior was analyzed in morphologically normal zebrafish between 11:00-a.m. and 5:00-p.m. under light conditions. At 5 dpf, zebrafish from each group were individually transferred to 48-well plates containing 500 μl of EM per well. After acclimation under light conditions for 30 min, movement was recorded for 5 min using a DanioVision (Noldus Information Technology, Wageningen, The Netherlands). Total distance moved was analyzed using an EthoVision XT 12 software (Noldus Information Technology) and calculated as the average distance moved per zebrafish per minute. Each test was independently repeated three times. For survival analysis, sibling controls, DMSO-treated *HuC*:mtFUS larvae, and OGS-MA114-treated *HuC*:mtFUS larvae were monitored daily from 1 to15 dpf. Dead larvae were counted and removed at each observation time point. Survival probability was calculated and plotted as Kaplan–Meier survival curves.

### TUNEL assay and immunohistochemistry (IHC) analysis in zebrafish

To assess apoptotic cell death, 11-dpf zebrafish were anesthetized with MS222 (200 mg/L; Sigma-Aldrich) and fixed in 4% paraformaldehyde (PFA). Fixed zebrafish were embedded in 1.5% agar blocks containing 5% sucrose, equilibrated in 30% sucrose, and cryosectioned at a thickness of 10 μm using a cryostat microtome (CM1860; Leica, Nussloch, Germany).

TUNEL assay combined with immunofluorescence staining was performed on zebrafish sections. Briefly, sections were dried, re-fixed with 1% PFA, washed with PBS, and permeabilized with 0.1 mM citric acid and 1% Triton X-100 for 2 min at 4°C. Sections were then incubated with TUNEL reaction mixture from the In Situ Cell Death Detection Kit, TMR red (Cat. No. 12156792910; Roche, Basel, Switzerland), for 1.5 h at 37°C. After washing and blocking with 2.5% sheep serum and 2.5% BSA, sections were incubated overnight at 4°C with mouse anti-Islet1 antibody (1:200; Developmental Studies Hybridoma Bank, Iowa City, IA, USA), followed by incubation with Alexa Fluor 647-conjugated goat anti-mouse IgG (1:1,000; Molecular Probes, Eugene, OR, USA). Fluorescence images were acquired using an A1 laser-scanning confocal microscope with 1-μm z-stacks and a Nikon Plan Apo 40×/1.25 objective (Nikon, Tokyo, Japan). TUNEL-positive cells were quantified in the spinal cord region.

### Statistical analysis

All quantitative data were obtained from at least three independent experiments and are presented as means ± standard error of the mean (SEM). Statistical analyses were performed using GraphPad Prism 10 software. Differences between two groups were analyzed using a two-tailed unpaired *t*-test, whereas multiple-group comparisons were performed using one-way analysis of variance (ANOVA) followed by Tukey’s multiple comparison or Tukey’s post hoc test, as appropriate. Survival analyses were performed using the log-rank test. A *p* value < 0.05 was considered statistically significant.

### Results

#### OGS-MA114 promotes mitophagy through PINK1-dependent pathway in SH-SY5Y cells

To identify novel cytoprotective mitophagy regulators, we screened a bioactive chemical library of approximately 11,000 compounds, including antiviral and autophagy-regulating compounds, in SH-SY5Y cells treated with the mitochondrial toxicant 1-methyl-4-phenylpyridinium (MPP^+^). MPP^+^ is a well-established mitochondrial toxicant that impairs complex I activity, leading to ATP depletion, oxidative stress, and mitochondrial injury; damaged mitochondria are subsequently targeted for mitophagic clearance as an adaptive quality-control response (Verma et al. 2020; Xu et al. 2023). From this screen, we identified 2-[1-[4-(4-chlorophenyl)−1,3-thiazol-2-yl]−5-hydroxy-3-methyl-1H-pyrazol-4-yl]-N-(3-acetylphenyl)acetamide (C₂₃H₁₉ClN₄O₃S), referred to as OGS-MA114, as the most potent suppressor of MPP^+^-induced cell death (Figure 1(A) and (B)). We next confirmed the cytoprotective effect of OGS-MA114 against MPP^+^-induced toxicity. As shown in Figure 1(C), exposure to MPP^+^ markedly reduced cell viability, whereas treatment with OGS-MA114 effectively attenuated MPP^+^-induced cytotoxicity and restored cell viability (Figure 1(C)). These results indicate that OGS-MA114 exerts a protective effect against MPP^+^-induced neuronal cell damage.

**Figure 1.**
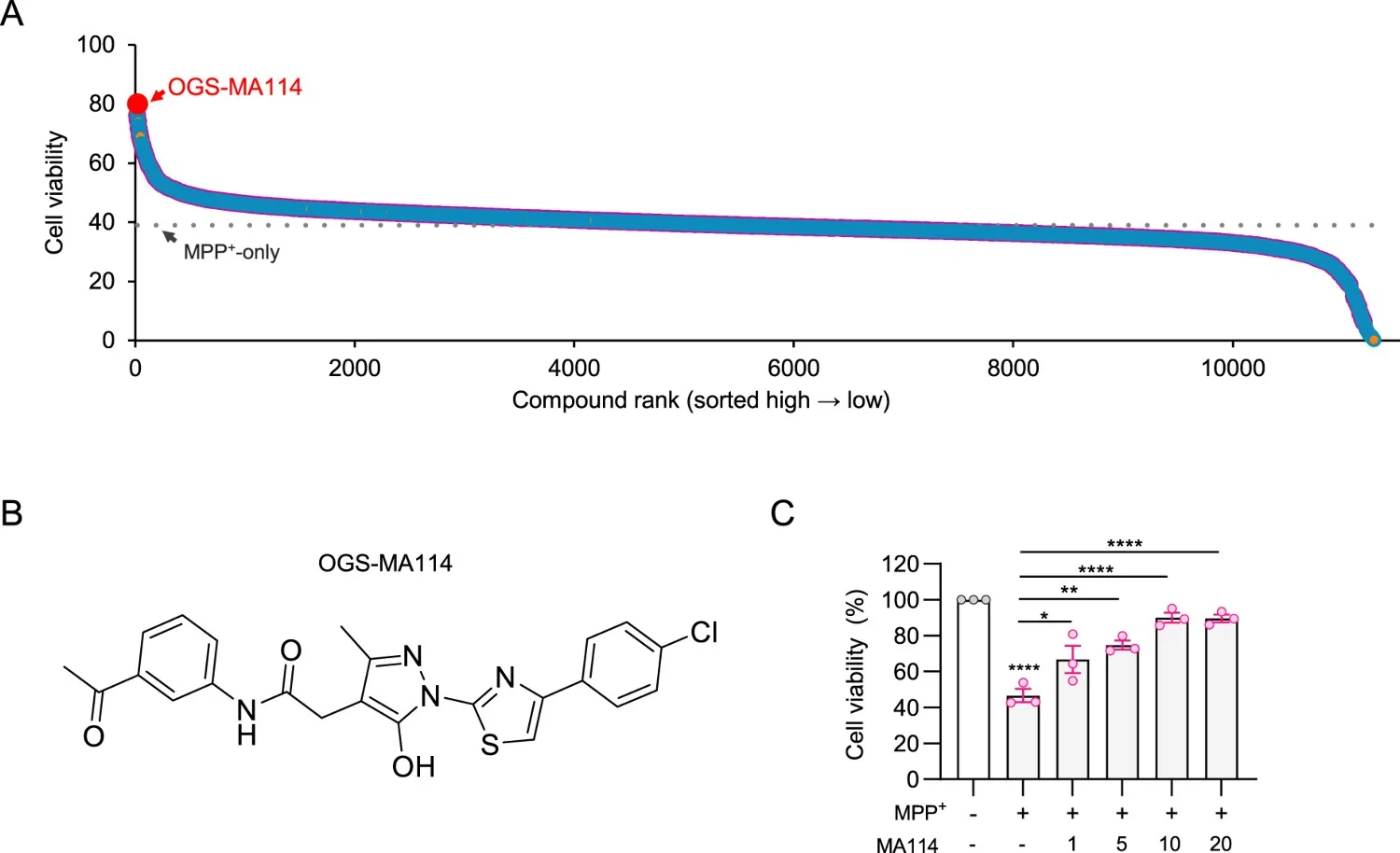
Identification of OGS-MA114 as a neuroprotective compound from a high-throughput cell viability screen. (A) Waterfall plot showing cell viability for 11,323 compounds screened in SH-SY5Y cells against MPP^+^-induced toxicity (see Materials and Methods for library composition and assay conditions). The gray dotted line denotes the MPP^+^ (2 mM)-only baseline (38.99 ± 4.33%). Of the compounds screened, 291 (2.57%) met the primary hit threshold and 41 (0.36%) were classified as strong hits. The assay yielded a mean Z′-factor of 0.912 ± 0.023 (range, 0.85–0.94; *n* = 36 plates). OGS-MA114 (red dot) was identified as one of the most potent compounds for rescuing of MPP^+^-induced cell death. (B) Chemical structure of OGS-MA114 (2-{1-[4-(4-chlorophenyl)-1,3-thiazol-2-yl]-5-hydroxy-3-methyl-1H-pyrazol-4-yl}-N-(3-acetylphenyl)acetamide; C₂₃H₁₉ClN₄O₃S; MW = 466.94). (C) SH-SY5Y cells were treated with OGS-MA114 at concentrations ranging from 1 to 20 μM in the presence or absence of MPP^+^ (2 mM) and cell viability was assessed using the CCK-8 assay. Statistical significance was determined by one-way ANOVA followed by Tukey’s multiple comparison test (*p < 0.05; **p < 0.01; ****p < 0.001).

We next examined whether OGS-MA114 affects mitophagy. As shown in Figure 2(A) and (B), treatment with low-dose CCCP (5 μM) alone induced only modest LC3-II accumulation and mitophagic flux in SH-SY5Y cells. In contrast, co-treatment with OGS-MA114 (20 μM) and CCCP (5 μM) significantly increased LC3-II accumulation and robustly enhanced mitophagic flux (Figure 2(A) and (B)). As the PINK1/Parkin pathway is a central mitochondrial quality-control mechanism responsible for the selective elimination of damaged mitochondria, we investigated whether this pathway contributes to the effect of OGS-MA114. Notably, OGS-MA114 markedly increased levels of Ser65-phosphorylated ubiquitin and PINK1 in SH-SY5Y cells (Figure 2(C)–(E)), indicating activation of PINK1-dependent mitochondrial ubiquitin signaling. In addition, OGS-MA114 promoted the recruitment of cytosolic Parkin to mitochondria (Figure 2(F)).

**Figure 2.**
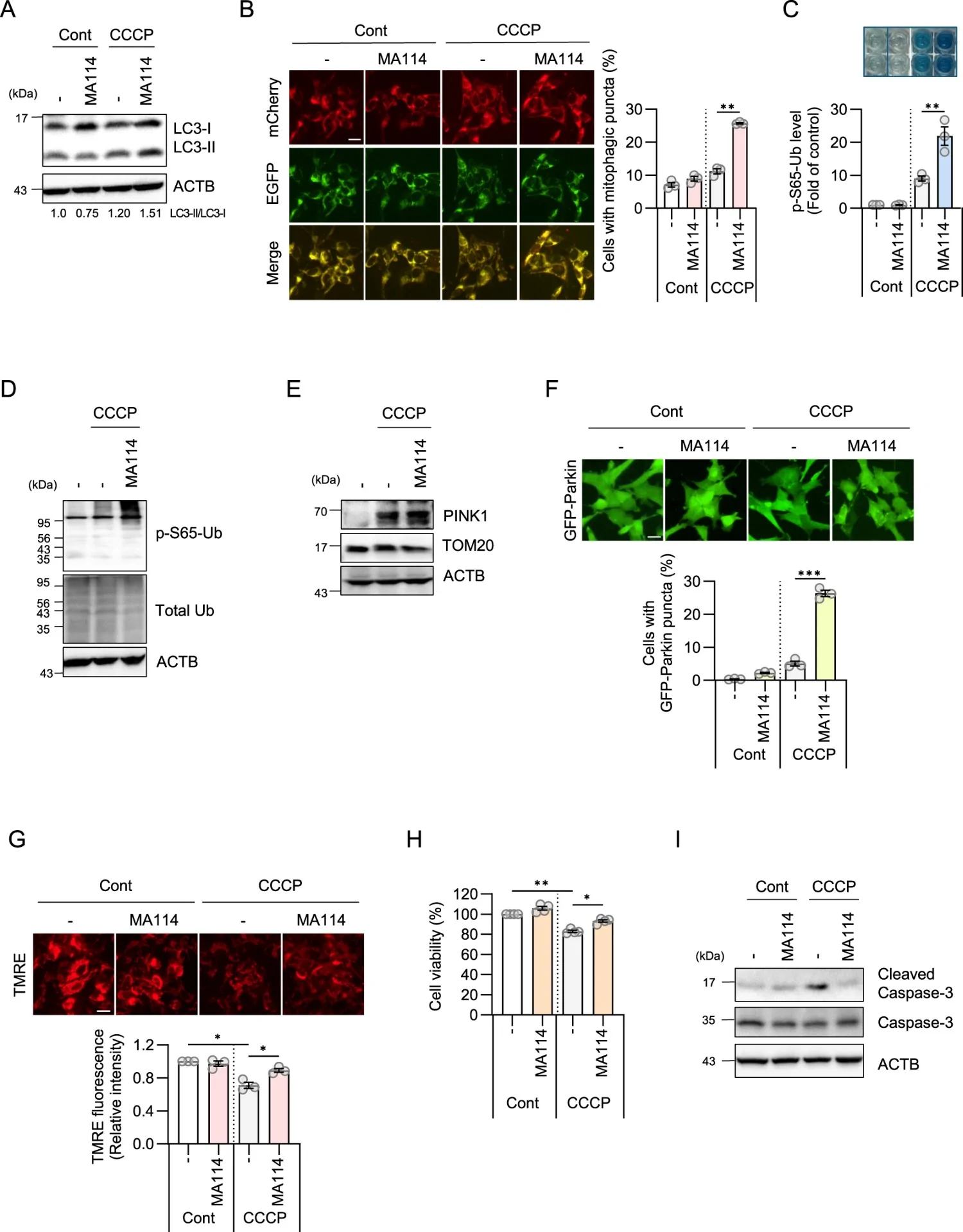
OGS-MA114 restores mitochondrial function under oxidative stress in SH-SY5Y cells. (A, B) SH-SY5Y cells were treated with OGS-MA114 (MA114, 20 μM) in the presence or absence of CCCP (5 μM) for 24 h. (A) LC3 protein levels were analyzed by Western blotting to evaluate autophagy-related responses. (B) Mitophagy flux was assessed using the Mito-QC reporter system, and fluorescence images were acquired using an Operetta CLS high-content imaging system (Revvity, Shelton, CT, USA) at 20× magnification. (C-F) SH-SY5Y cells were treated with OGS-MA114 (MA114, 20 μM) in the presence or absence of CCCP (5 μM) for 6 h. (C) Phospho-ubiquitin (Ser65) expression levels in cell lysates were quantified by ELISA assay. (D, E) Phospho-ubiquitin (Ser65) and PINK1 levels were analyzed by Western blotting. (F) Parkin translocation was assessed by fluorescence imaging using an Operetta CLS high-content imaging system (Revvity) at 20× magnification. (G-I) SH-SY5Y cells were treated with OGS-MA114 (MA114, 20 μM) in the presence or absence of CCCP (5 μM) for 24 h. (G) Mitochondrial membrane potential was assessed by TMRE staining. TMRE fluorescence intensity was measured using a Synergy H1 microplate reader (BioTek) at excitation/emission wavelengths of 550/580 nm, and fluorescence images were acquired using an Operetta CLS high-content imaging system at 20× magnification. (H) Cell viability was measured using CCK-8 assay kit. (I) Active caspase-3 and total caspase-3 protein levels were analyzed by Western blotting. Scale bar, 20 μm. Data are presented as the mean ± SEM from at least three independent experiments. Statistical significance was determined by one-way ANOVA followed by Tukey’s multiple comparison test (*p < 0.05; **p < 0.01; ***p < 0.005).

Oxidative stress-induced mitochondrial damage is a major pathogenic feature of neurodegenerative disorders, including ALS and Parkinson’s disease (PD), and age-related impairment of mitophagy further exacerbates the accumulation of dysfunctional mitochondria and neuronal vulnerability (De Gaetano et al. 2021; Madruga et al. 2021; Hyung et al. 2025). To assess the effects of OGS-MA114 on mitochondrial function, SH-SY5Y cells were treated with CCCP in the presence or absence of OGS-MA114, and mitochondrial membrane potential was measured. As shown in Figure 2(G), treatment with CCCP induced mitochondrial membrane depolarization. To determine whether the OGS-MA114-mediated mitophagy enhancement was accompanied by cytoprotective effects, we further assessed cell viability and apoptosis-related signaling. OGS-MA114 significantly restored cell viability in CCCP-treated SH-SY5Y cells (Figure 2(H)), and markedly reduced cleaved caspase-3 levels (Figure 2(I)), suggesting that OGS-MA114 mitigates CCCP-induced cytotoxicity and apoptotic signaling. Together, these findings indicate that OGS-MA114 promotes mitophagy in SH-SY5Y cells at least in part through a PINK1-dependent pathway, and exerts cytoprotective effects.

#### OGS-MA114 enhances mitophagy and reduces FUS^P525L^-associated cellular toxicity in SH-SY5Y cells

*FUS* mutations such as *FUS*^P525L^ are strongly associated with an aggressive juvenile-onset form of ALS. Accumulating evidence suggests that ALS-linked *FUS* mutations are also associated with autophagy defects, mitochondrial dysfunction, and neurotoxicity (Chua et al. 2022; Moens et al. 2025). We next examined whether OGS-MA114 could alleviate neuronal toxicity induced by the ALS-associated FUS^P525L^ mutant. In SH-SY5Y cells expressing FUS^WT^ or FUS^P525L^, OGS-MA114 increased LC3-II accumulation under low-dose CCCP-induced mitochondrial stress conditions; autophagic flux was further assessed in the presence of bafilomycin A1 (Figure 3(A)). OGS-MA114 also increased levels of Ser65-phosphorylated ubiquitin and PINK1, a marker of PINK1/Parkin-mediated mitophagy, with a stronger effect in FUS^P525L^-expressing cells than in FUS^WT^-expressing cells (Figure 3(B)–(D)). In addition, TMRE analysis showed that CCCP markedly reduced mitochondrial membrane potential in both FUS^WT^- and FUS^P525L^-expressing SH-SY5Y cells, whereas OGS-MA114 significantly restored mitochondrial membrane potential under these conditions (Figure 3(E)). Moreover, OGS-MA114 reduced FUS^P525L^- and CCCP-induced cell death, as indicated by restored cell viability and decreased cleaved caspase-3 levels (Figure 3(F) and (G)). Together, these results suggest that OGS-MA114 protects SH-SY5Y cells against FUS^P525L^-associated mitochondrial stress and cellular toxicity by promoting PINK1/Parkin-dependent mitophagy.

**Figure 3.**
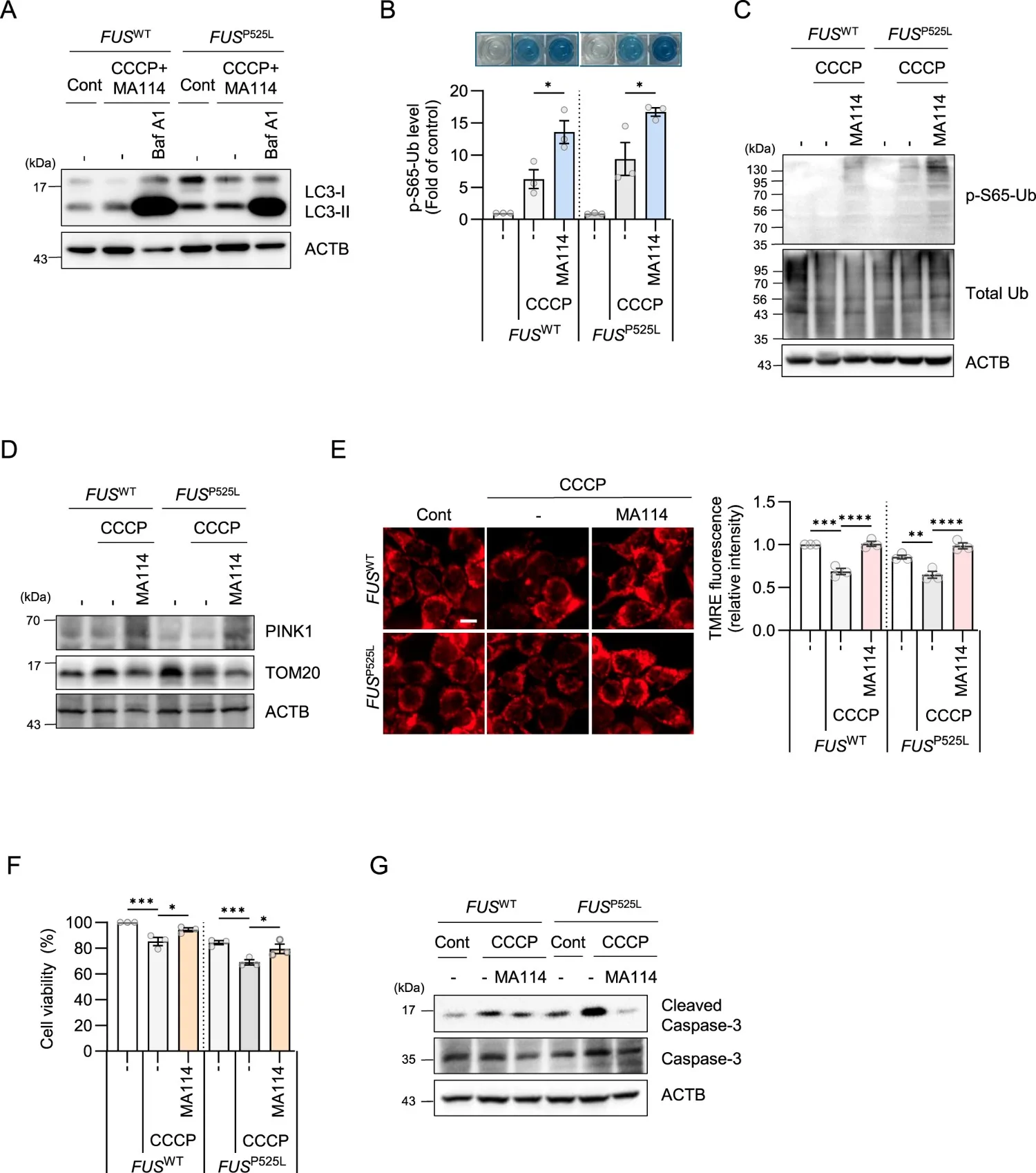
OGS-MA114 enhances mitophagy and reduces FUS^P525L^-associated cellular toxicity in SH-SY5Y cells (A) SH-SY5Y/GFP-FUS^WT^ and SH-SY5Y/GFP-FUS^P525L^ cells were treated with CCCP (5 μM) and OGS-MA 114 (MA114, 20 μM), in the presence or absence of bafilomycin A1 (Baf A1, 20 nM) for 24 h. LC3 protein levels were analyzed by Western blotting to assess autophagic flux. (B-G) SH-SY5Y/GFP-FUS^WT^ and SH-SY5Y/GFP-FUS^P525L^ cells were treated with CCCP (5 μM) in the presence or absence of OGS-MA114 (20 μM) for 24 h. (B) Phospho-ubiquitin (Ser65) levels in cell lysates were measured using an ELISA kit. (C, D) Phospho-ubiquitin (Ser65) and PINK1 levels were analyzed by Western blotting. (E) Mitochondrial membrane potential was assessed by TMRE staining. Fluorescence intensity was measured using a Synergy H1 microplate reader (BioTek) at excitation/emission wavelengths of 550/580 nm, and fluorescence images were acquired using an Operetta CLS high-content imaging system (Revvity) at 20 × magnification. (F) Cell viability was determined by CCK-8 assay. (G) Cleaved caspase-3 and total caspase-3 protein levels were analyzed by Western blotting. Scale bar, 20 μm. Data are presented as the mean ± SEM from at least three independent experiments. Statistical significance was determined by one-way ANOVA followed by Tukey’s multiple comparison test (*p < 0.05; **p < 0.01; ***p < 0.005; ****p < 0.001).

#### OGS-MA114 ameliorates ALS-associated phenotypes in zebrafish model expressing the FUS^R521C^ mutant

To further investigate the in vivo effects of OGS-MA114, we used a zebrafish model expressing the ALS-associated FUS^R521C^ mutant. *FUS* mutations, including R521C, have been associated with motor dysfunction, cytoplasmic mislocalization, and neurodegenerative phenotypes in vertebrate and zebrafish models (Acosta et al. 2014; Moens et al. 2025). In this model, *HuC*:mtFUS zebrafish exhibited a marked reduction in locomotor activity compared with sibling controls. OGS-MA114 treatment significantly restored locomotor activity in *HuC*:mtFUS zebrafish, indicating that OGS-MA114 alleviates FUS^R521C^-induced motor impairment in vivo (Figure 4(A)).

**Figure 4.**
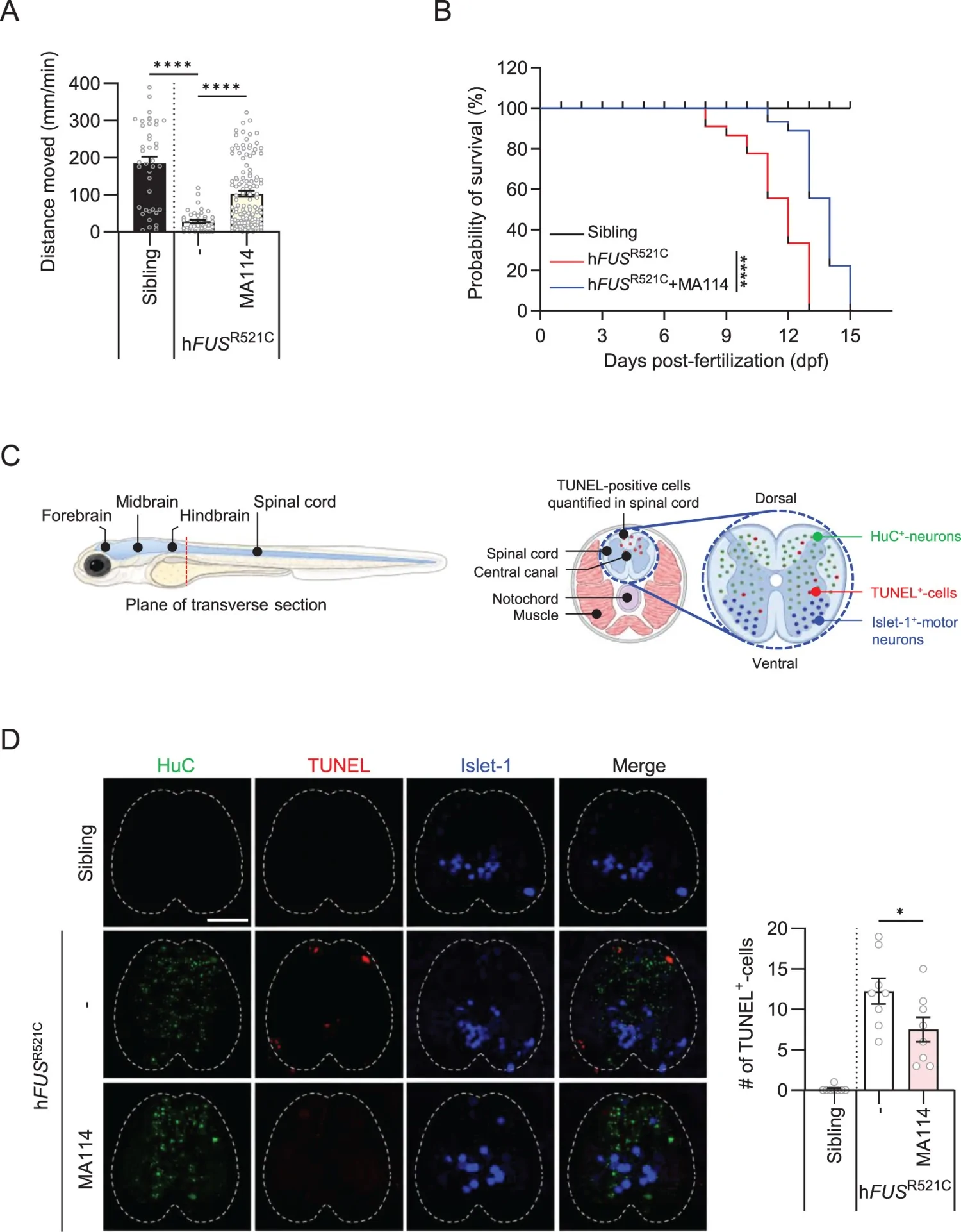
OGS-MA114 improves motor function and survival and reduces cell death in FUS^R521C^ zebrafish (A) Locomotor activity of sibling controls, DMSO-treated *HuC*:mtFUS larvae, and OGS-MA114-treated *HuC*:mtFUS larvae at 5 dpf, measured as distance moved per minute. n = 8–24 larvae per group. (B) Kaplan–Meier survival curves of sibling controls, DMSO-treated *HuC*:mtFUS larvae, and OGS-MA114-treated *HuC*:mtFUS larvae from 1 to 15 dpf. n = 30–45 larvae per group. (C, D) The approximate boundaries of the spinal cord are indicated by dashed outlines. The accompanying anatomical schematic illustrates the dorsal–ventral orientation of the transverse spinal cord section and identifies the signals analyzed using the same color coding as the experimental images (C). TUNEL-positive cells within the spinal cord were quantified from five sections per larva. (D) Representative confocal fluorescence images and quantification of TUNEL-positive cells in 10-μm-thick transverse spinal cord cryosections from 11-dpf larvae. Neurons expressing hFUS^R521C^ were identified by ZsGreen fluorescence driven by the HuC neuronal promoter (green), whereas apoptotic cells and motor neurons were detected by TUNEL staining (red) and Islet-1 immunostaining (blue), respectively. Data are presented as means ± S.E.M. Statistical significance was determined by one-way ANOVA followed by Tukey’s post hoc test for panel A, the log-rank test for panel B, and a two-tailed unpaired t-test for panel D. *p < 0.05 and ****p < 0.0001 vs. DMSO-treated *HuC*:mtFUS. Scale bar, 50 μm.

We next assessed whether this functional improvement was accompanied by increased survival. *HuC*:mtFUS zebrafish showed a progressive decline in survival probability, whereas OGS-MA114 treatment significantly improved survival compared with DMSO-treated *HuC*:mtFUS zebrafish (Figure 4(B)). *HuC*:mtFUS zebrafish also exhibited increased apoptotic cell death in the spinal cord, as shown by the elevated number of TUNEL-positive cells. In contrast, OGS-MA114 treatment significantly reduced the number of TUNEL-positive cells in the spinal cord (Figure 4(C) and (D)). Collectively, these results indicate that OGS-MA114 ameliorates ALS-associated phenotypes in vivo and support its therapeutic potential as a mitophagy-activating neuroprotective compound.

## Discussion

In this study, we identified OGS-MA114 as a mitophagy-activating compound that suppresses mitochondrial toxin- and mutant FUS-associated toxicity. Increased levels of LC3, phospho-ubiquitin, and PINK1-dependent Parkin translocation support the interpretation that OGS-MA114 acts primarily through the PINK1/Parkin axis. This pathway is particularly relevant to ALS, in which mitochondrial damage can contribute to ROS production, membrane depolarization, and bioenergetic failure (Youle and Narendra 2011; Evans and Holzbaur 2019; Chua et al. 2022). Consistent with this mechanism, OGS-MA114 preserved mitochondrial membrane potential, and attenuated cell death in mutant FUS-expressing cells, suggesting that its protective effects are associated with restoration of mitochondrial homeostasis rather than a nonspecific anti-cell death effect (Evans and Holzbaur 2019).

In addition to the PINK1/Parkin pathway, receptor-mediated pathways involving BNIP3/NIX and FUNDC1 can promote mitochondrial clearance under stress conditions and may act in parallel with or in concert with, ubiquitin-dependent mitophagy (Liu et al. 2012; Yamashita et al. 2024). Because OGS-MA114 increased both autophagic and mitophagic flux, further studies are needed to clarify whether it selectively activates the PINK1-pathway or more broadly enhances mitochondrial quality control. For the in vivo experiments, we used a zebrafish FUS^R521C^ model rather than the FUS^P525L^ model used in the cellular system, because preliminary overexpression of the FUS^P525L^ in zebrafish caused excessive lethality, limiting its utility for pharmacologic evaluationt (Acosta et al. 2014; Moens et al. 2025). Therefore, future studies should evaluate OGS-MA114 across a broader range of FUS variants, including P525L, R521H/C, and other FUS variants, to determine whether its efficacy is associated with mutation severity or with shared mitochondrial stress phenotypes.

Transactive response DNA binding protein 43 kDa (TDP-43) is also closely linked to ALS pathogenesis, and its pathology is strongly associated with mitochondrial dysfunction. Previous studies have shown that enhancement of autophagy can promote TDP-43 clearance and ameliorate disease-associated phenotypes in neuronal and mouse models (Barmada et al. 2014; Huang et al. 2020). If OGS-MA114 also improves mitochondrial function and cell survival in TDP-43 models, it would strengthen the possibility that the compound targets a convergent organelle quality-control mechanism shared across ALS subtypes rather than through a FUS-specific mechanism (Chua et al. 2022).

From a medicinal chemistry perspective, the pyrazole-thiazole aryl-amide scaffold of OGS-MA114 contains heteroaromatic structural features commonly found in bioactive compounds, including kinase- and enzyme-modulating small molecules (Alam 2023; Sumran et al. 2024). Although the present findings do not identify a direct target, it raises the possibility that OGS-MA114 may influence upstream signaling involved in mitochondrial stress sensing, ubiquitin signaling, or autophagy initiation. To further develop OGS-MA114 as a lead compound, additional preclinical studies will be necessary to evaluate its safety, pharmacological properties, and therapeutic potential (Masimirembwa et al. 2003).

In conclusion, our findings suggest that OGS-MA114 is a potential mitophagy activator that ameliorates ALS pathology via a PINK1-dependent pathway.

## Supplementary Material

OGS MA114_Raw data_Revision 20260724.xlsx

OGS MA114_Original Image_Western blotting_Revision 20260724.pdf

## Data Availability

All data generated during this study are included in this article. Additional raw data are available from the corresponding author upon reasonable request.
