# Supplementary material for: OGS-MA114 ameliorates FUS mutant-associated ALS pathology by activating PINK1-dependent mitophagy
Source: Anim Cells Syst (Seoul). 2026 Nov 10;30(1):644–56. doi: 10.1080/19768354.2026.2736980 (PMC13757885; doi:10.1080/19768354.2026.2736980)

Full blot for Figure 2A

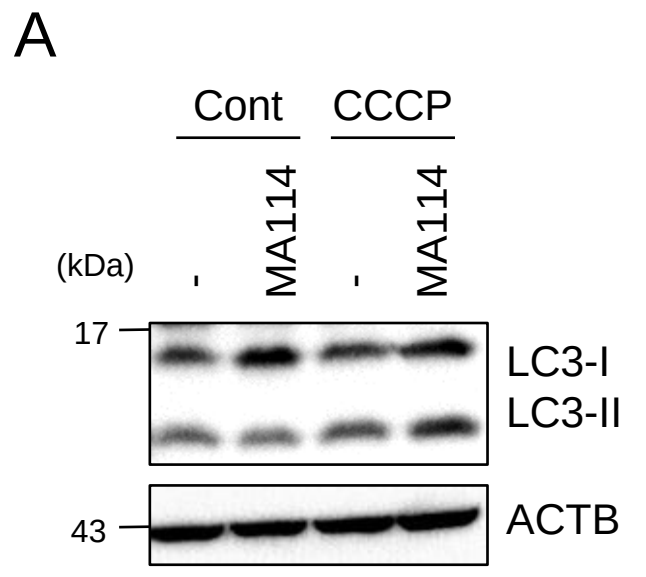

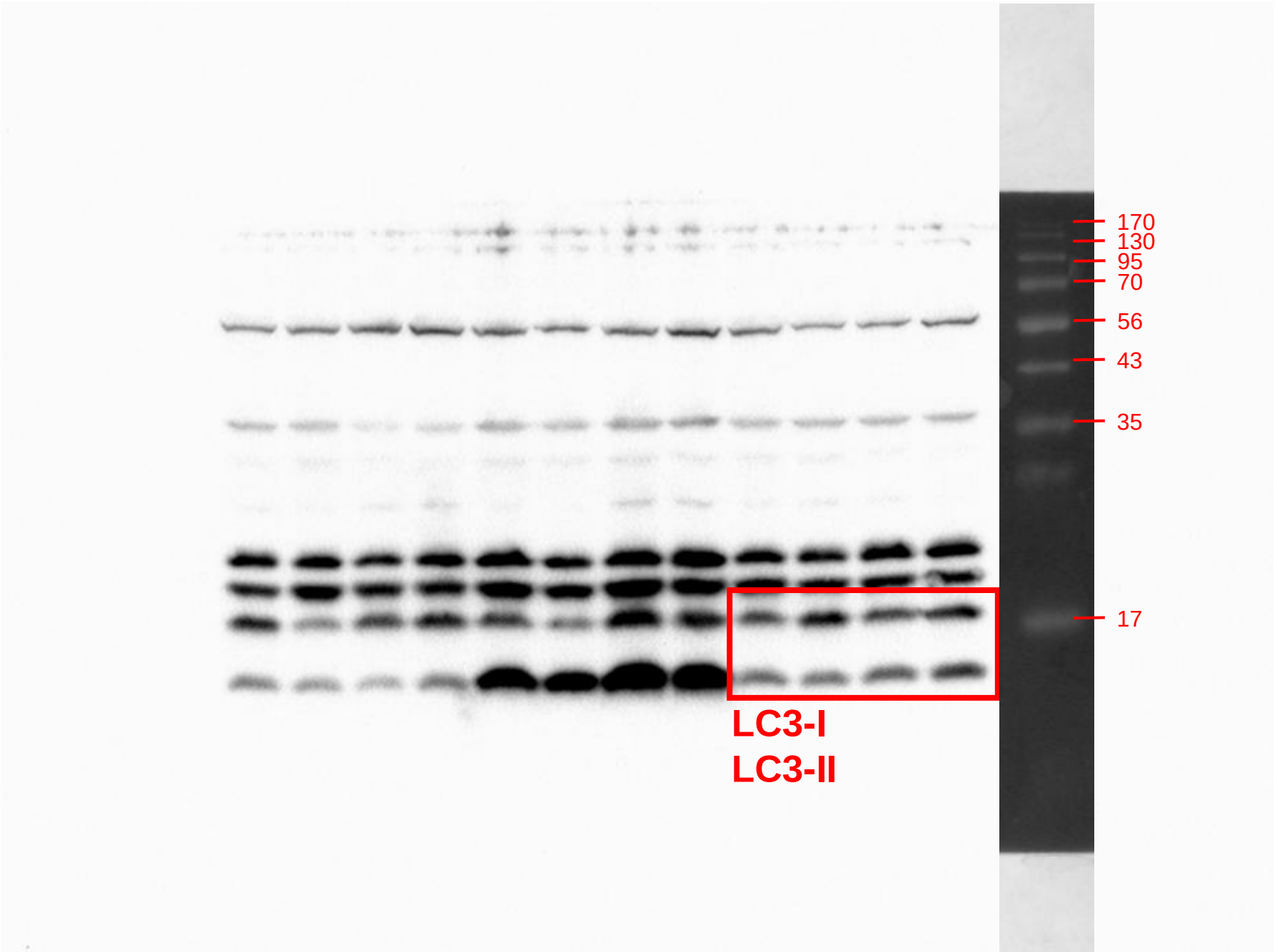

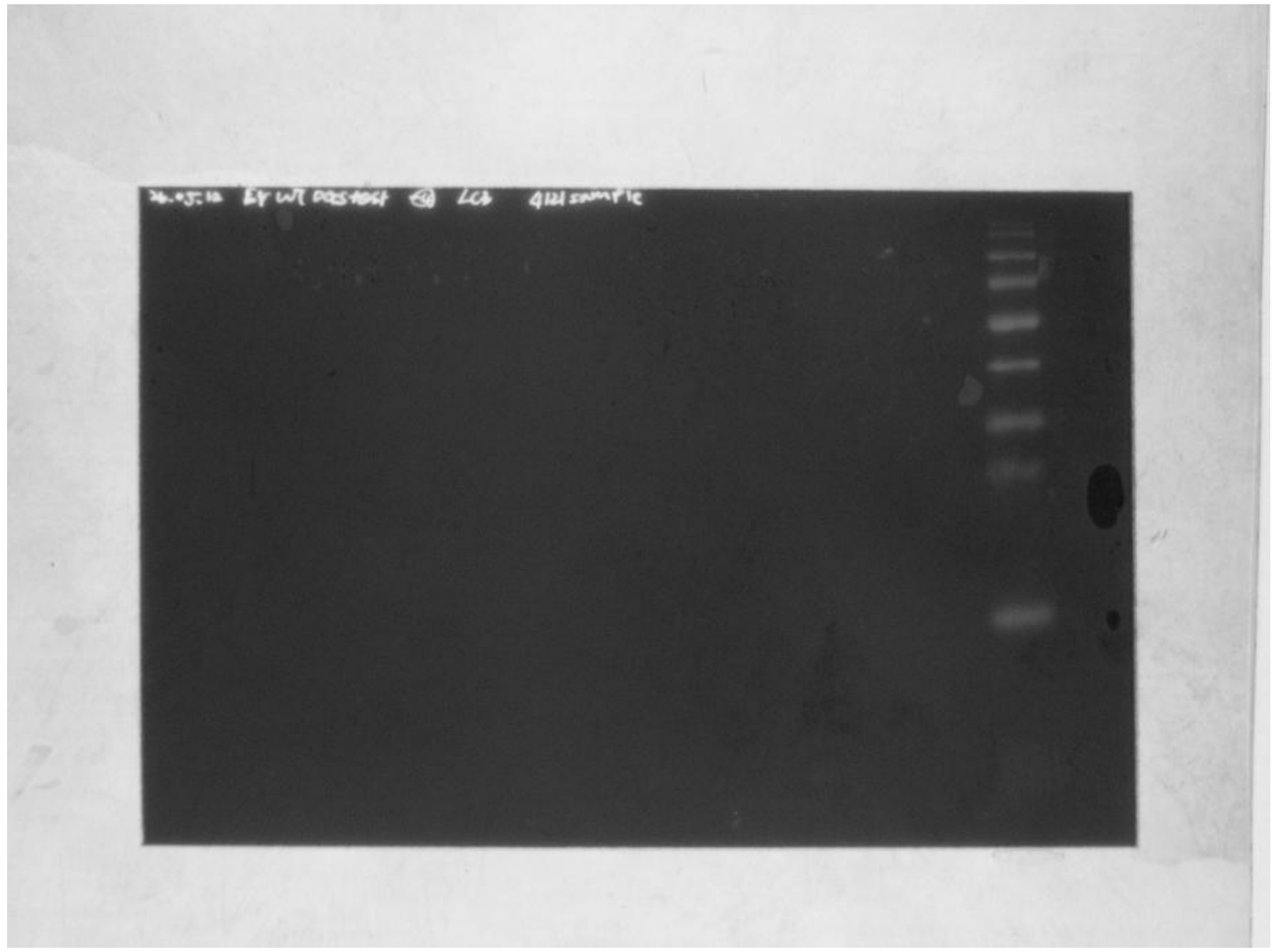

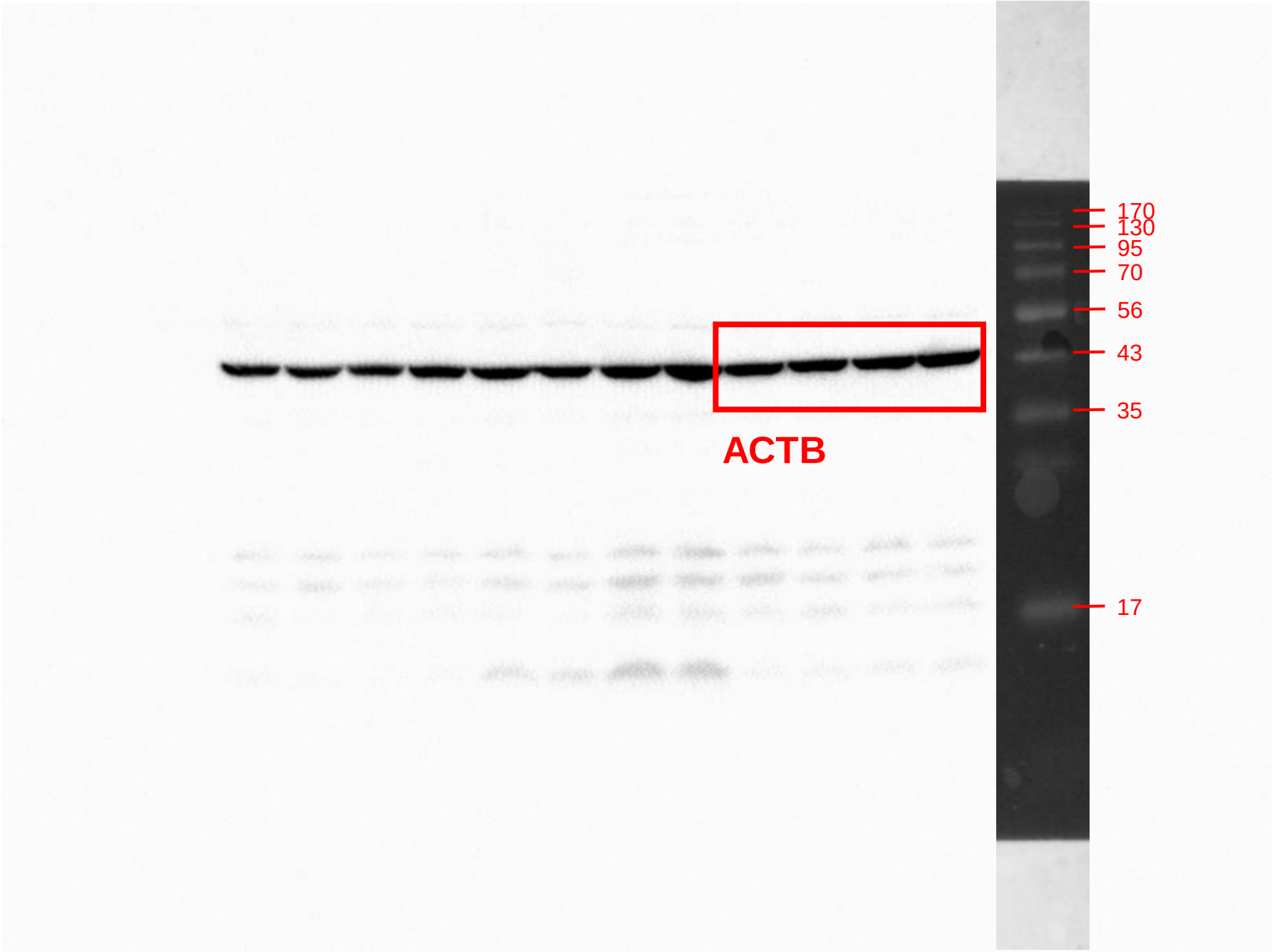

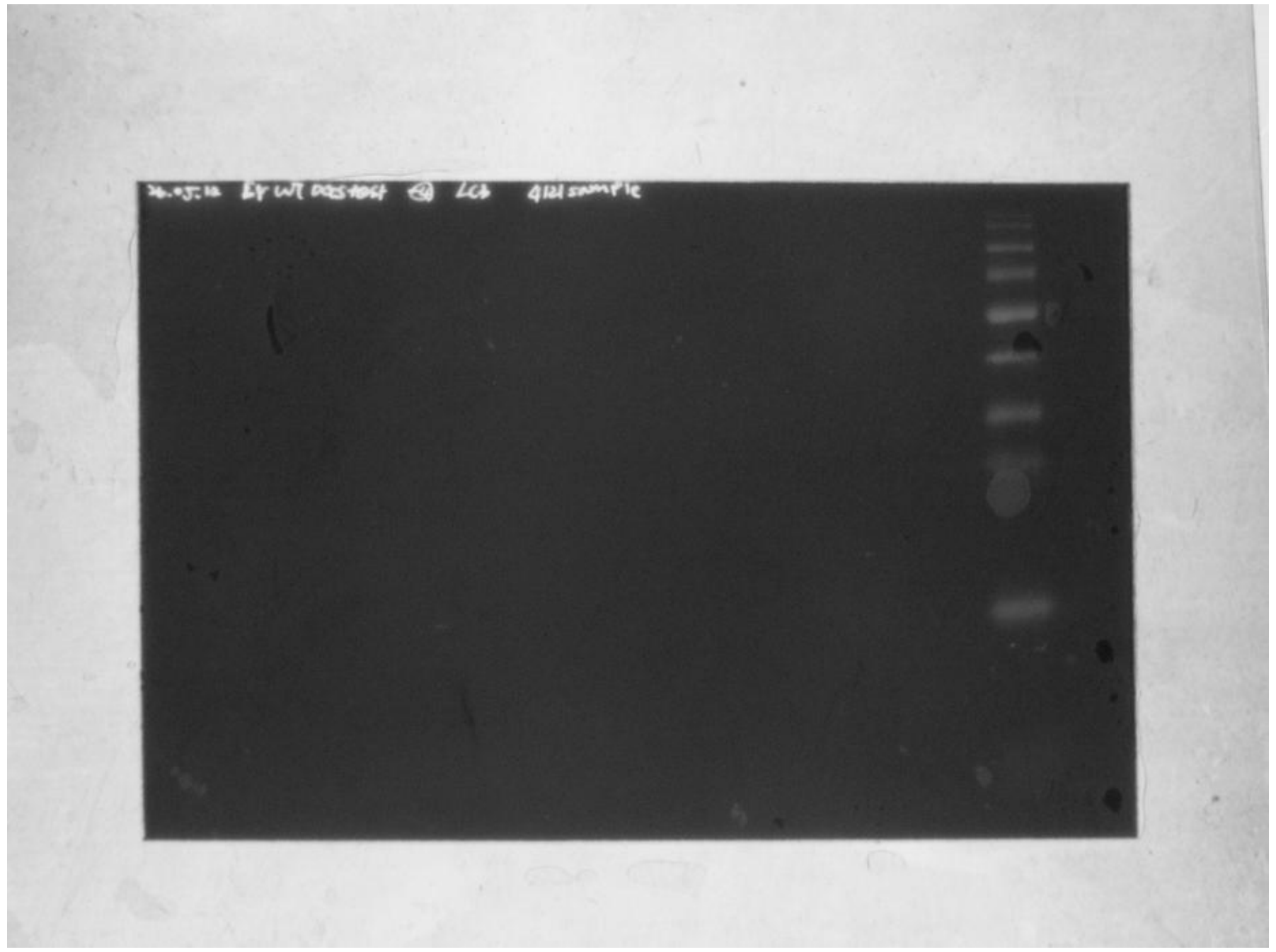

Full blot for Figure 2D

D

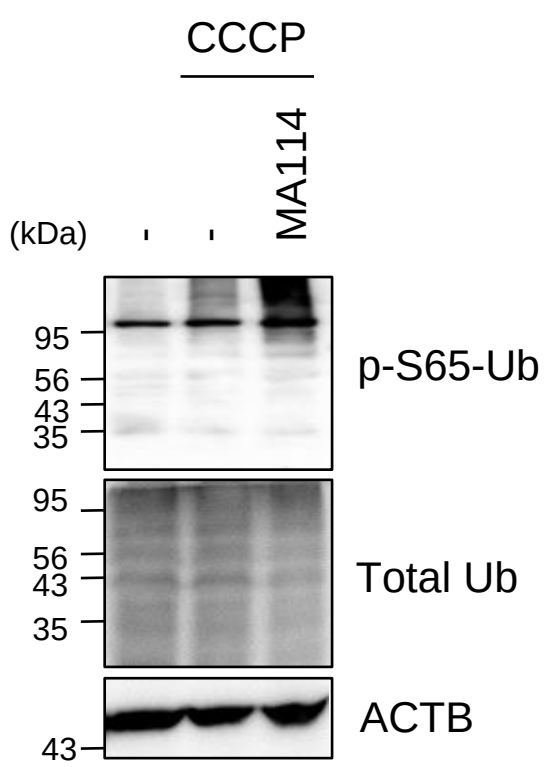

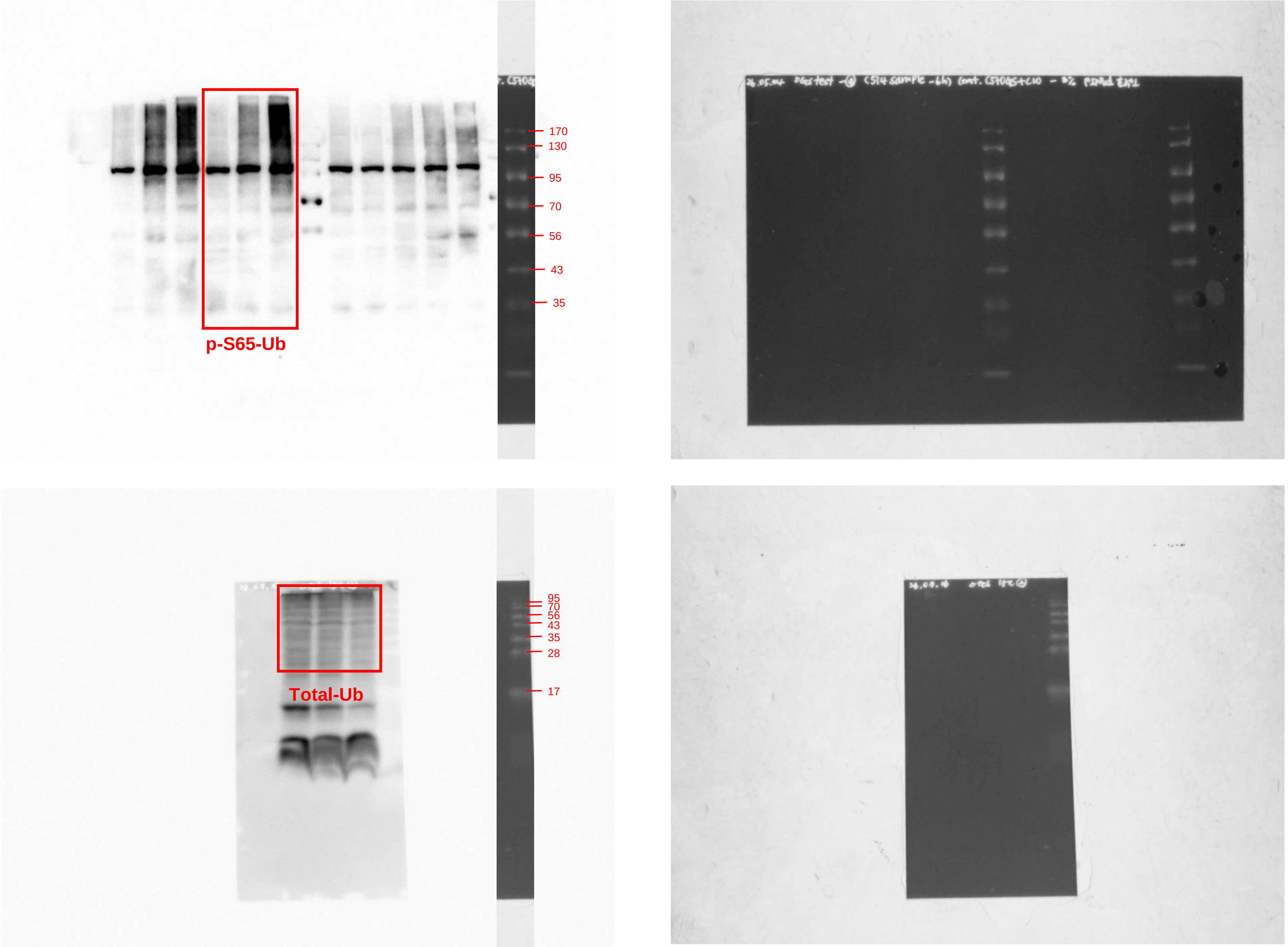

Full blot for Figure 2D

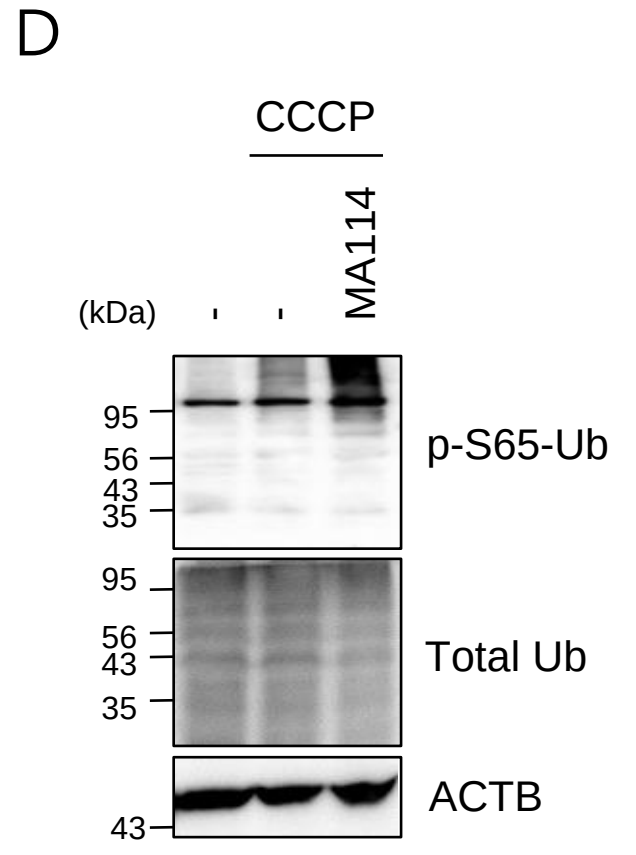

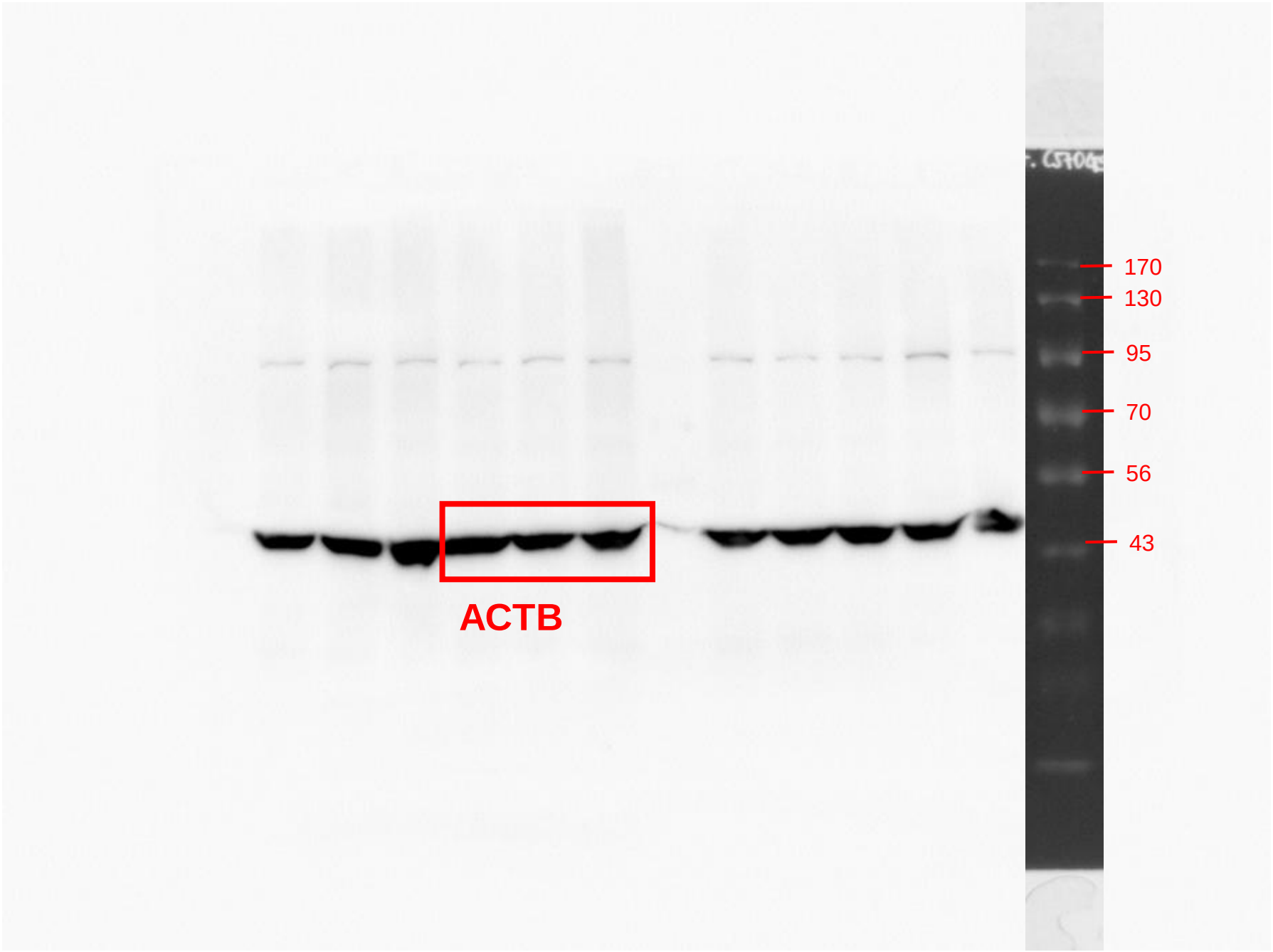

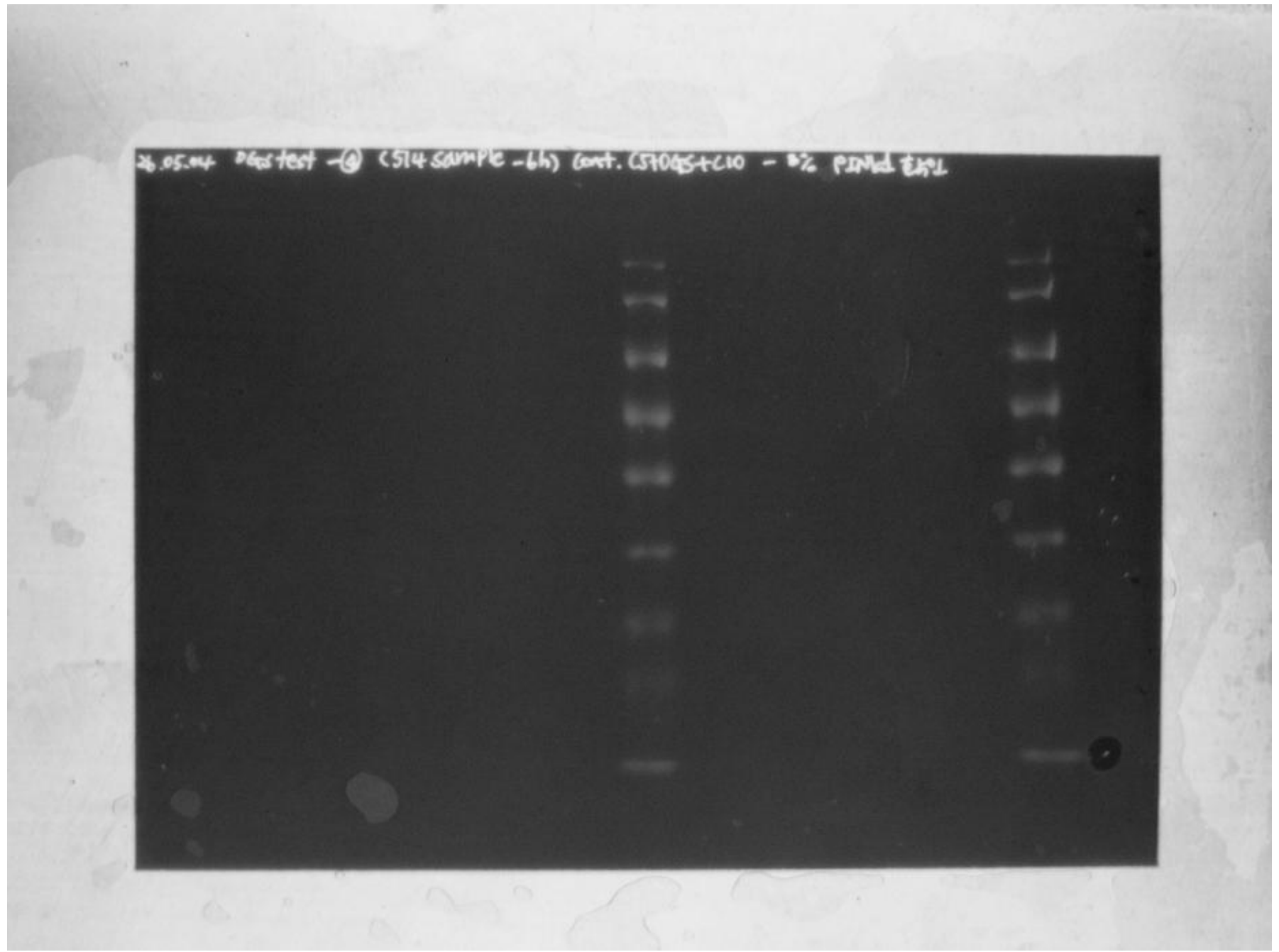

Full blot for Figure 2E

E

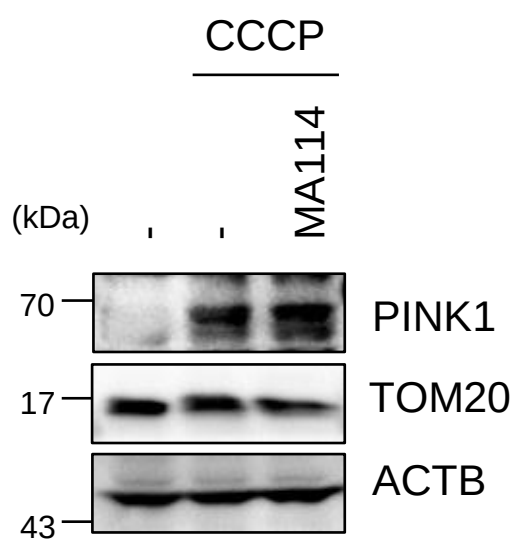

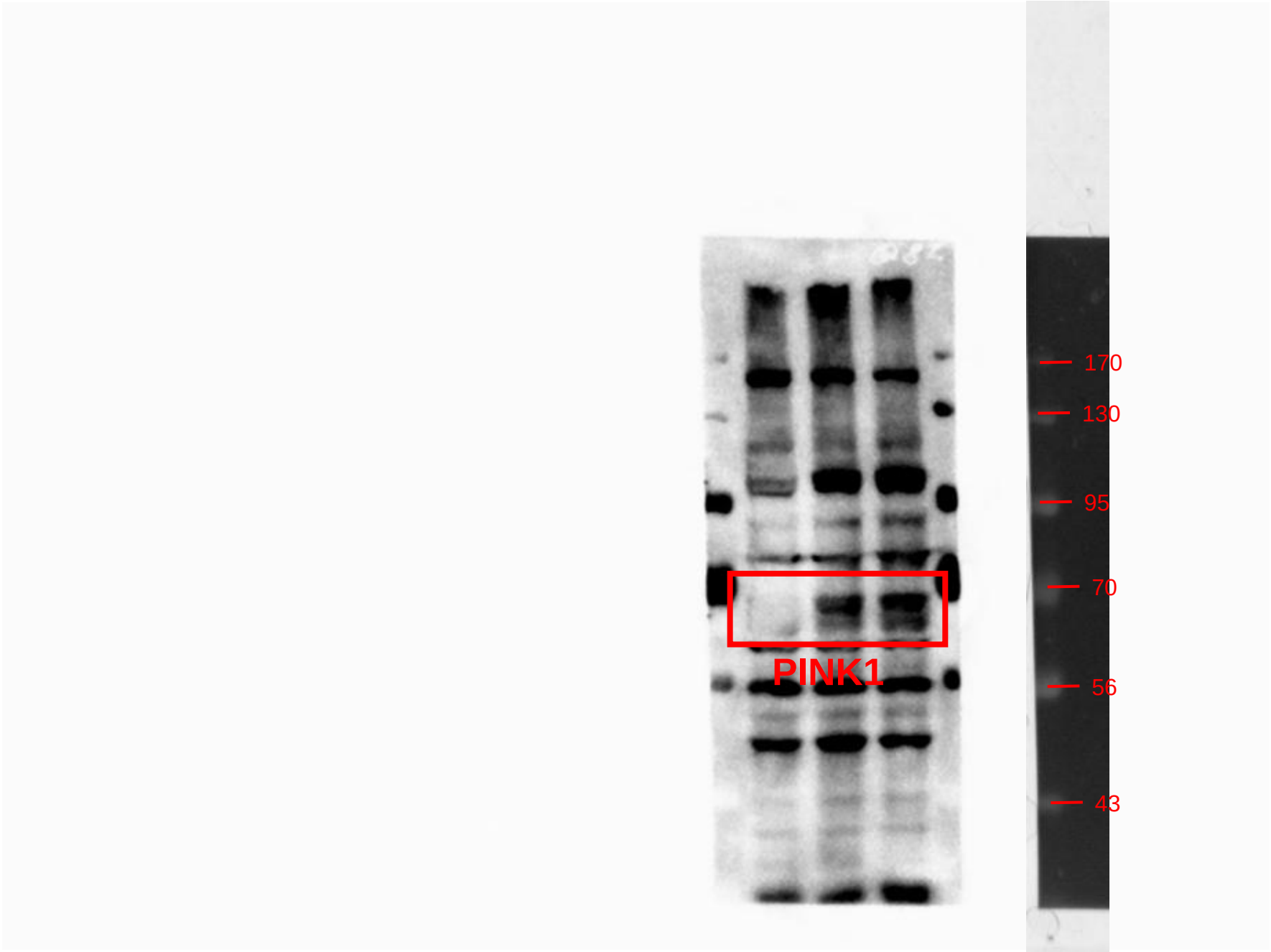

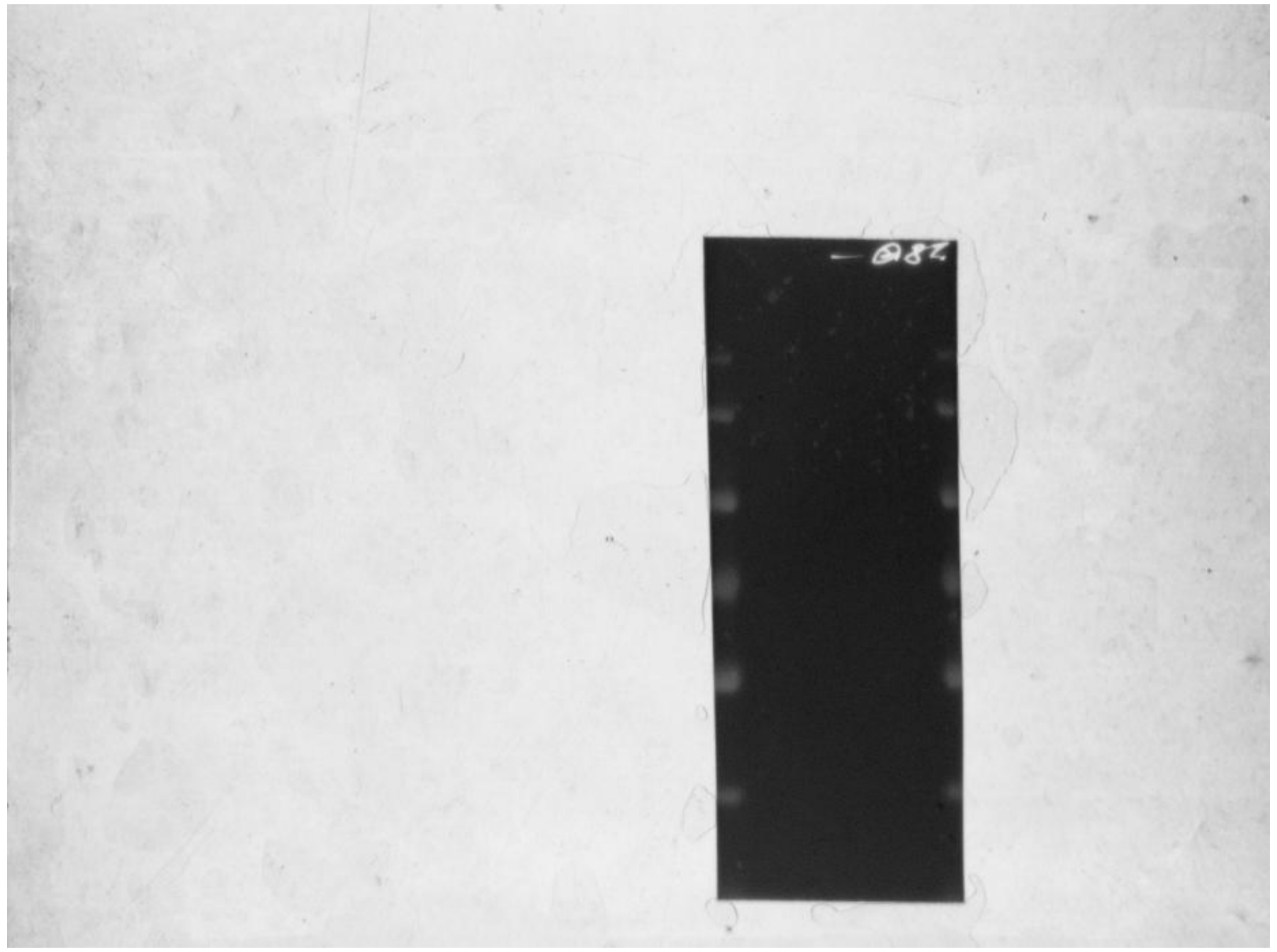

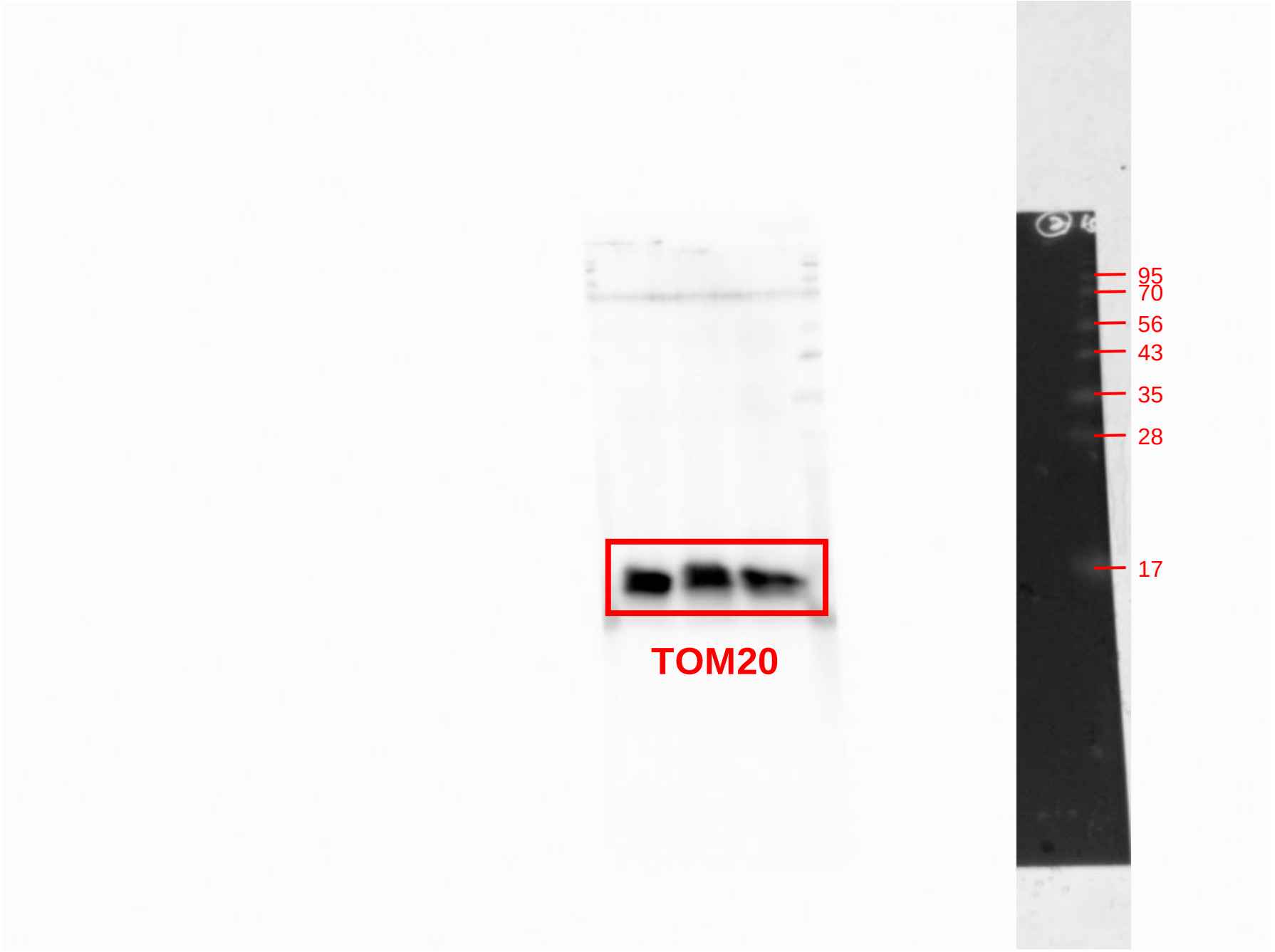

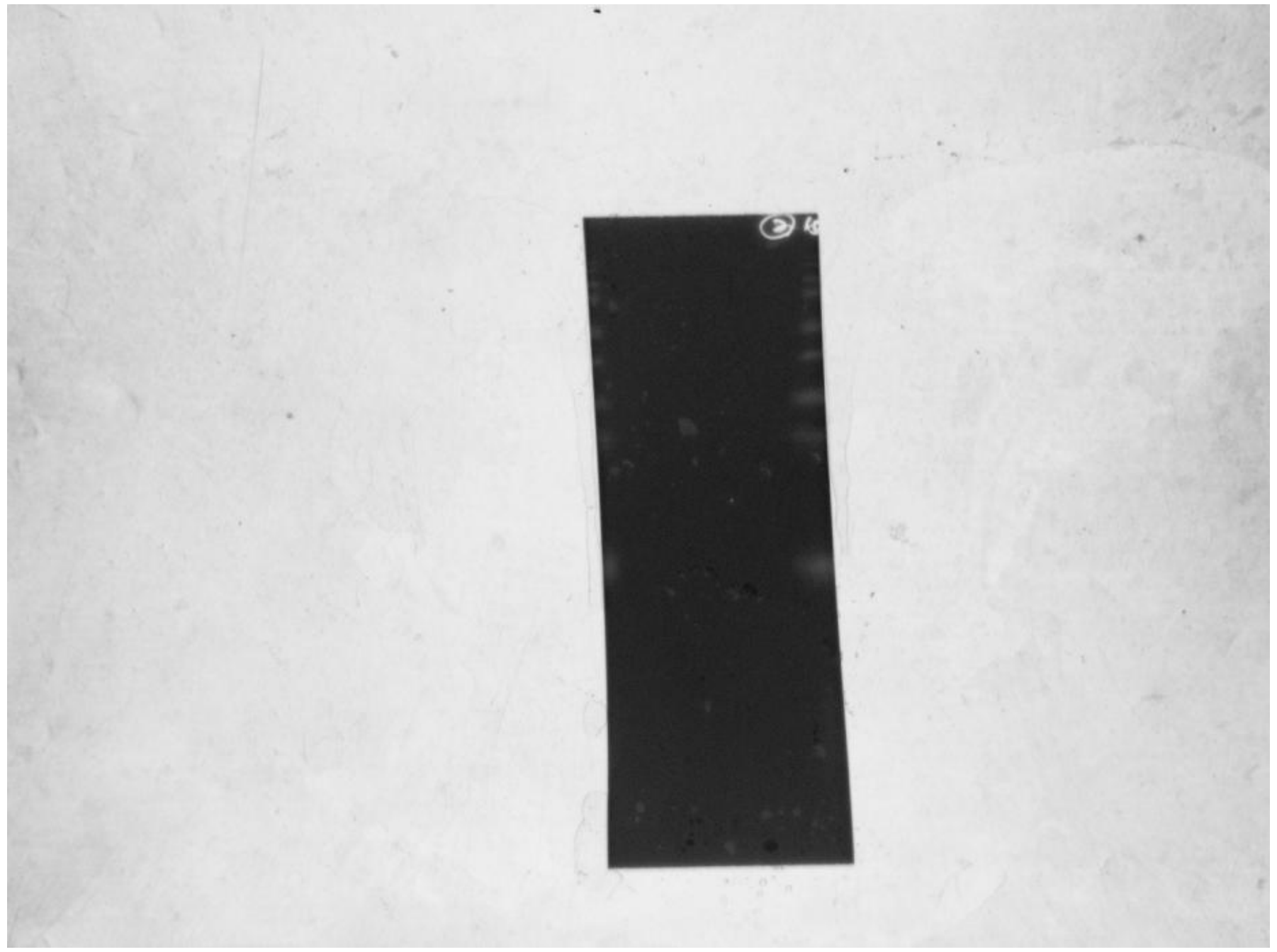

Full blot for Figure 2E

E

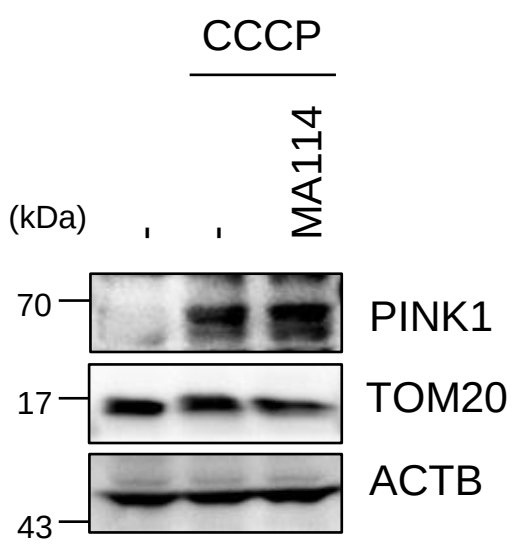

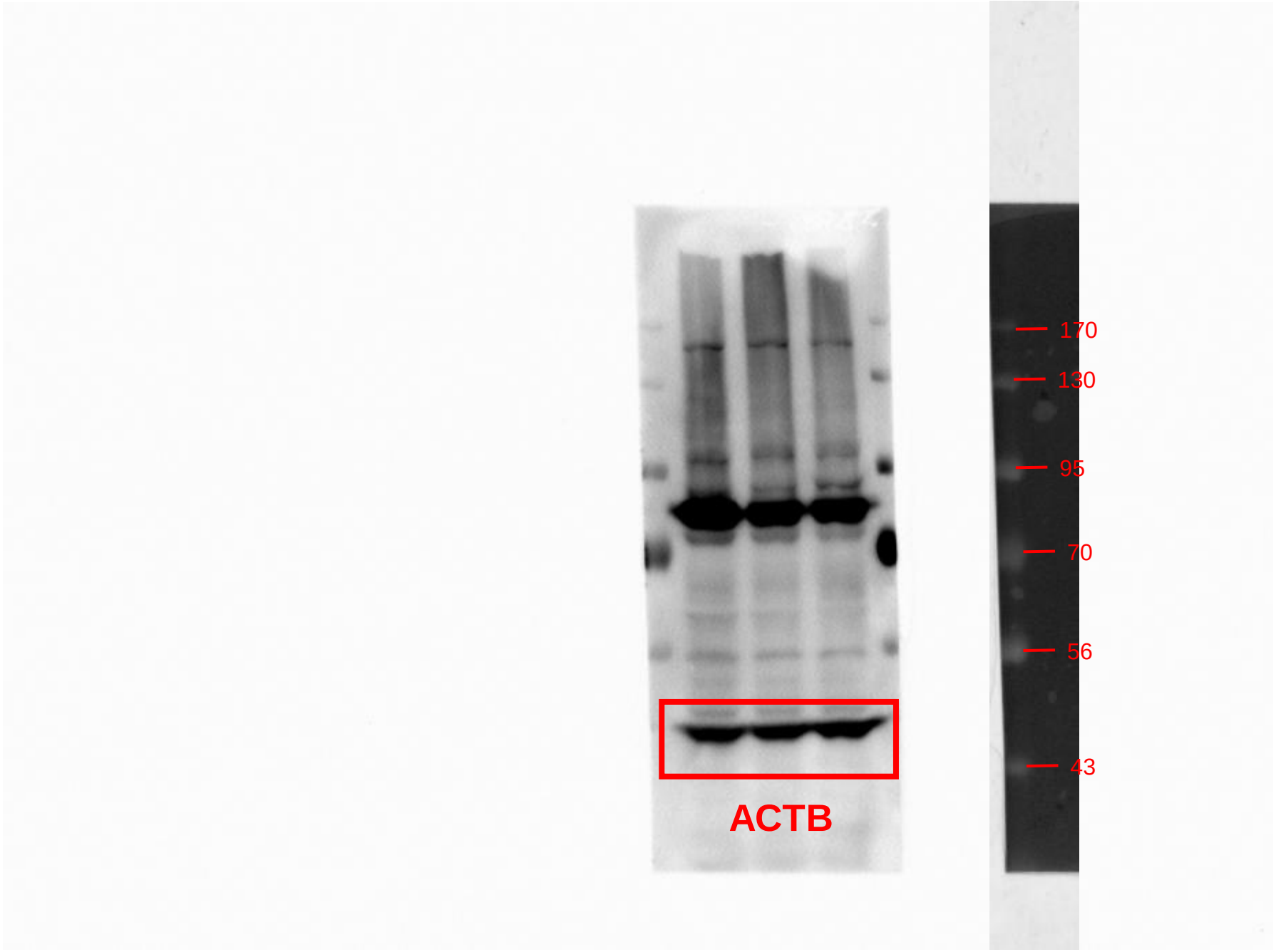

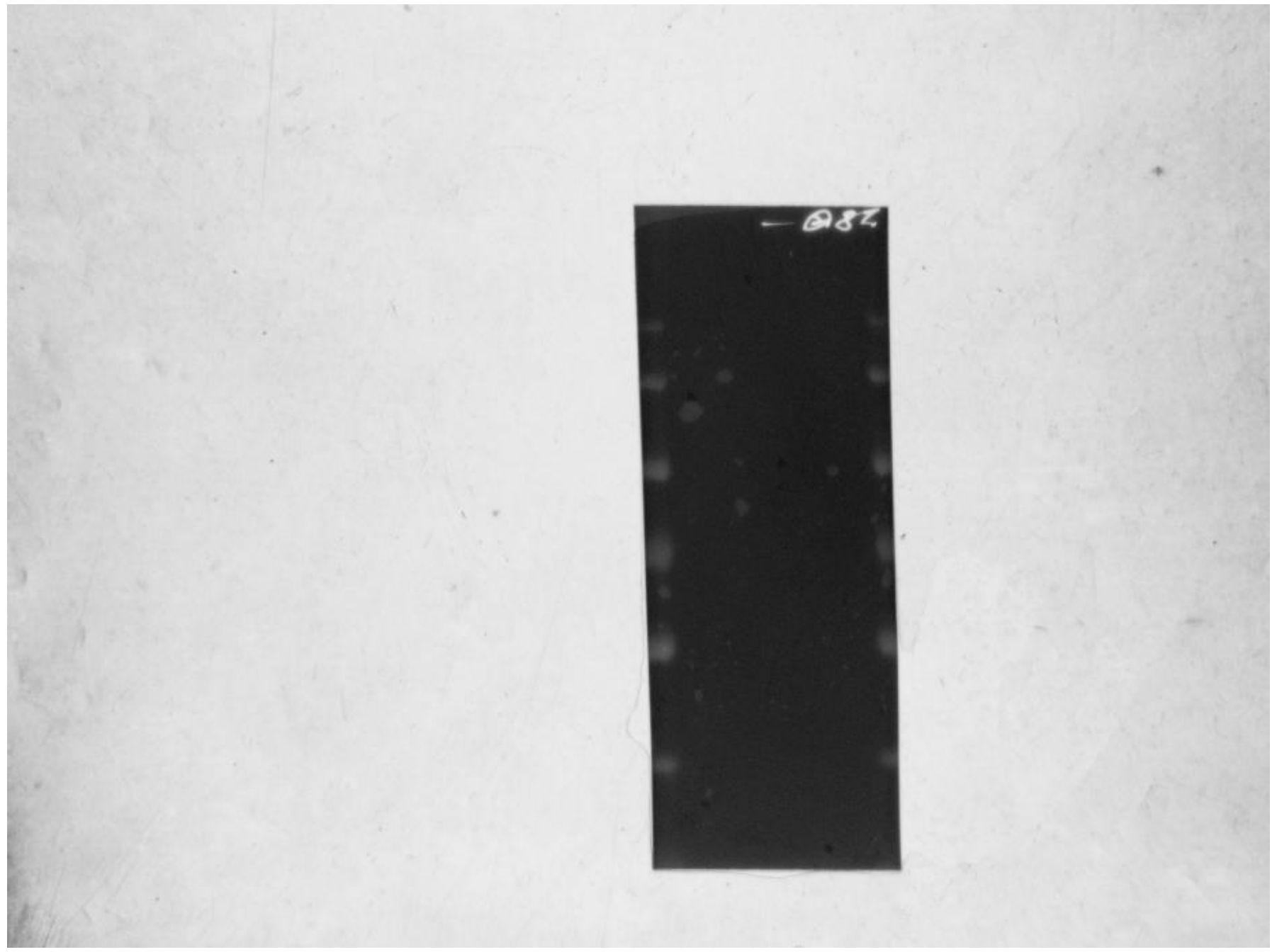

Full blot for Figure 2I

I

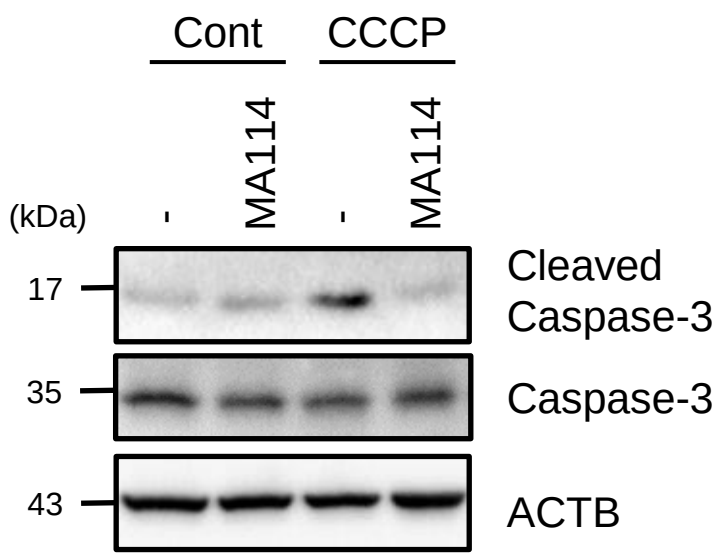

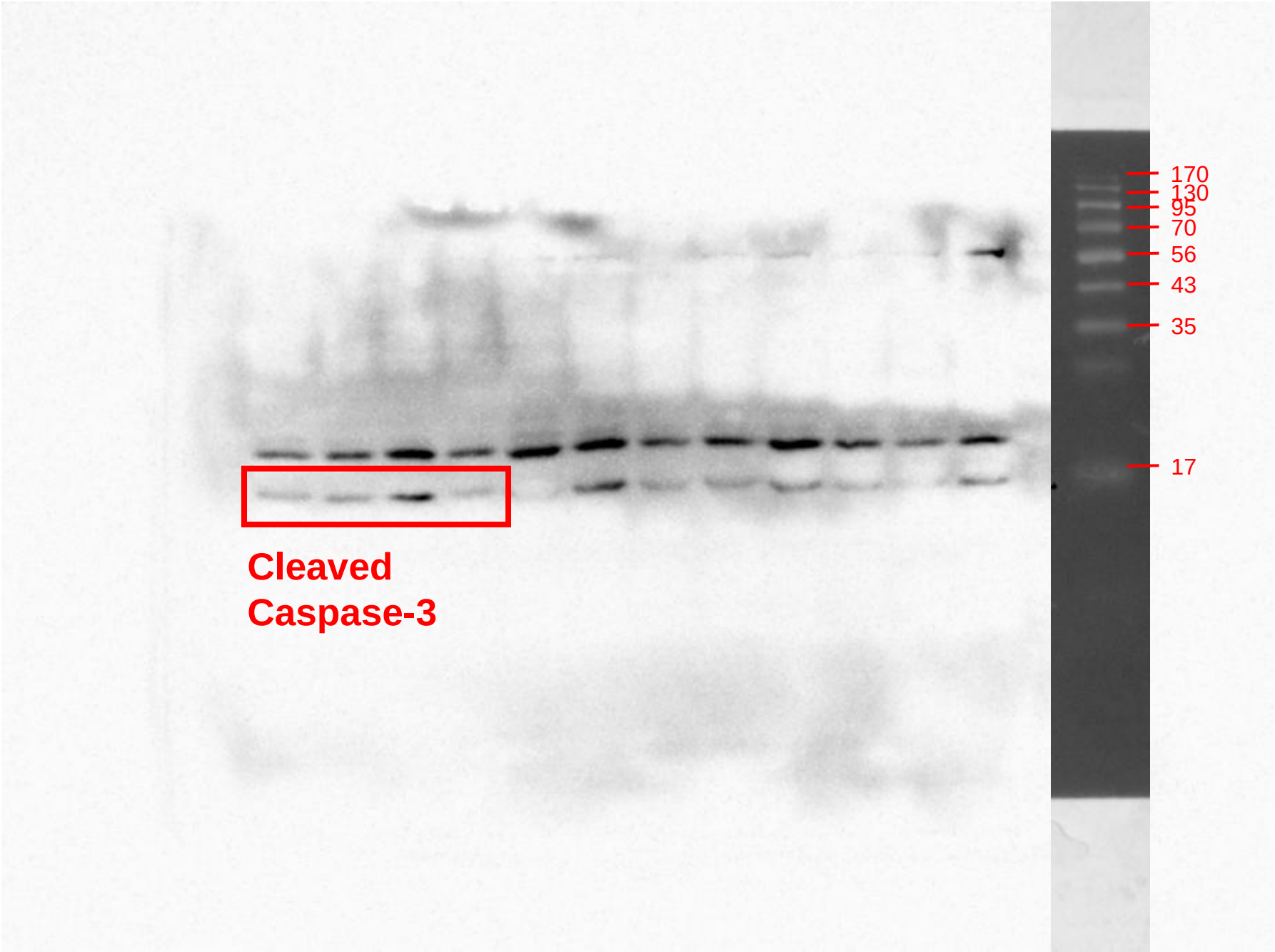

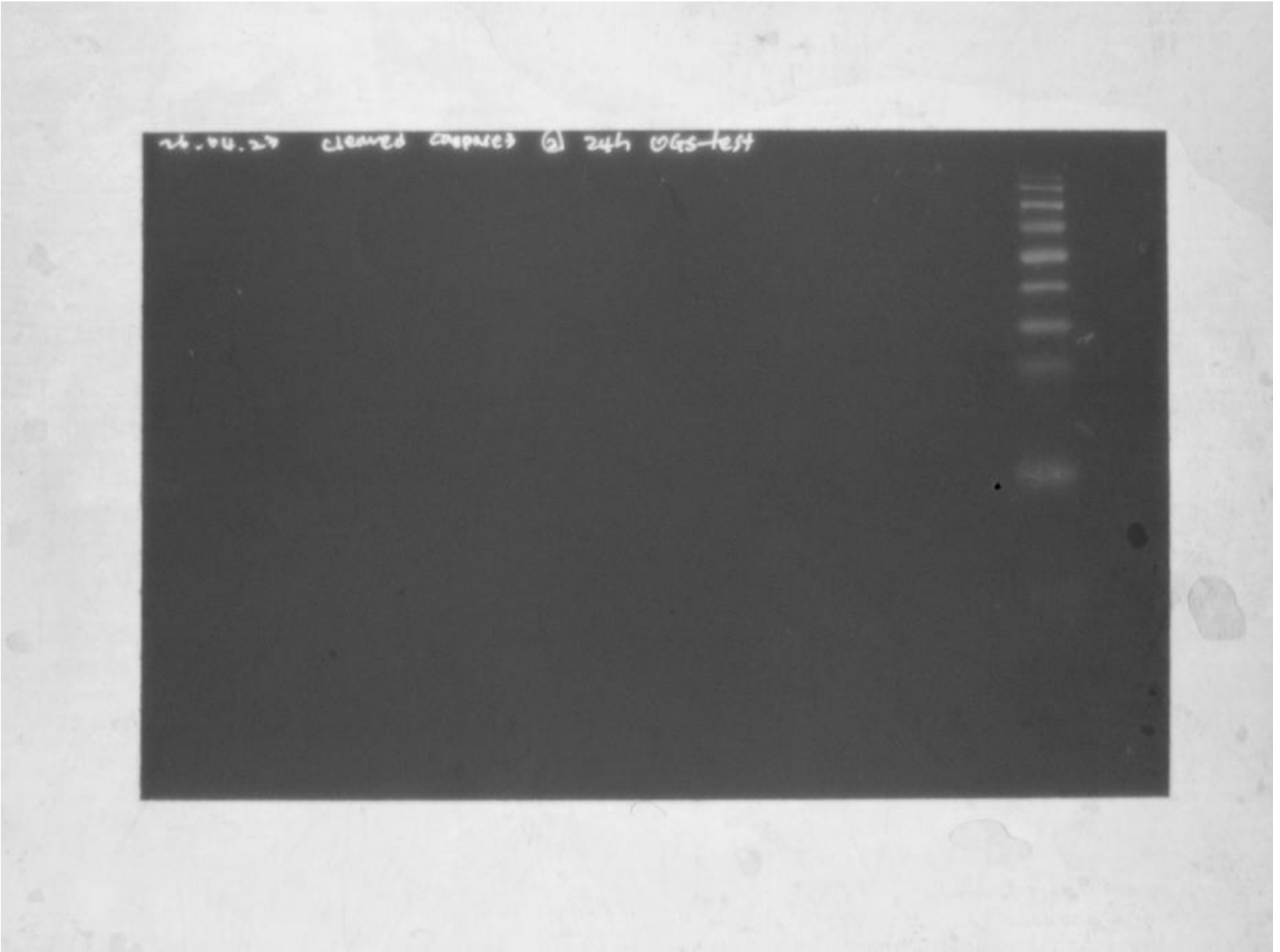

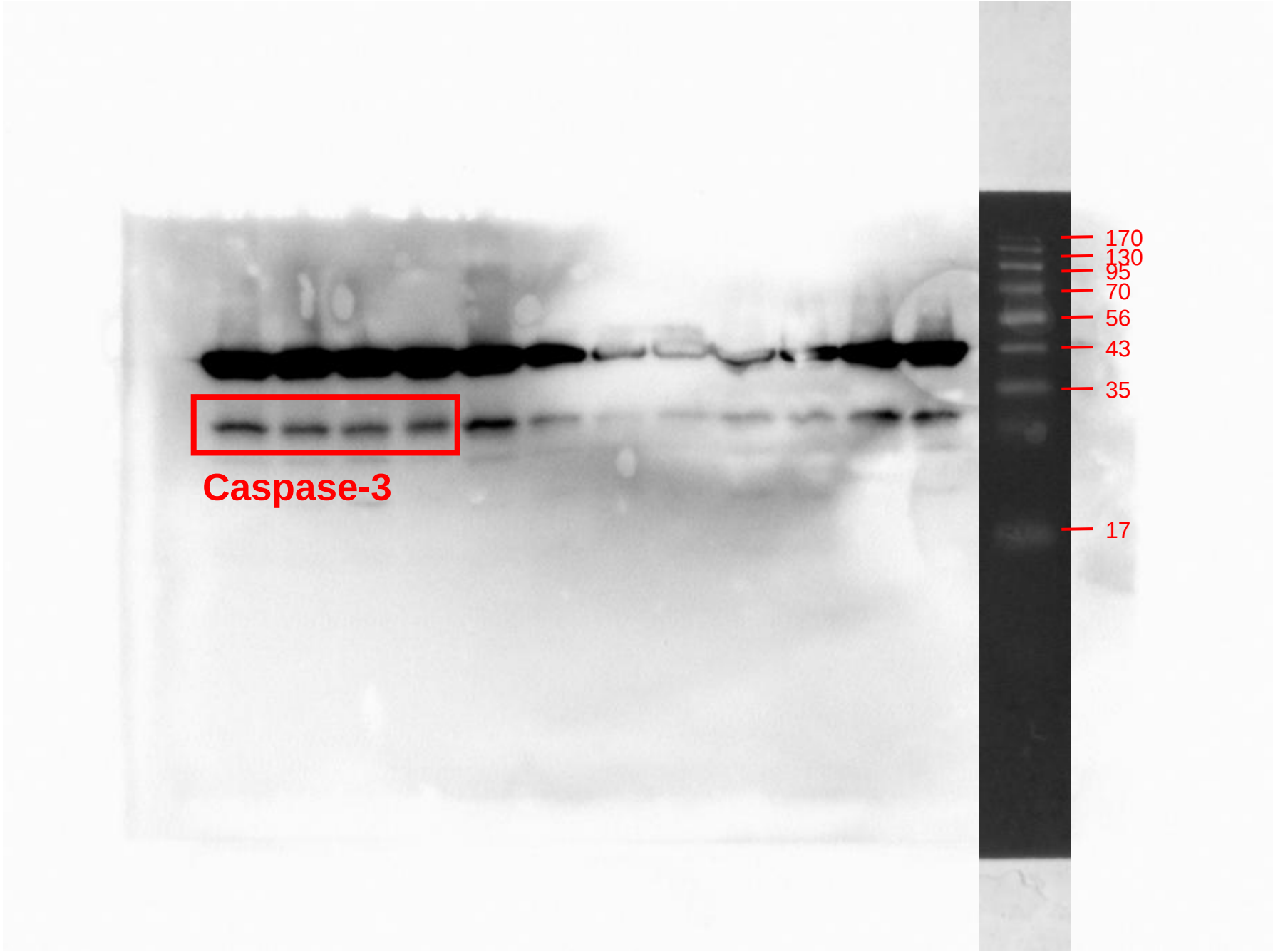

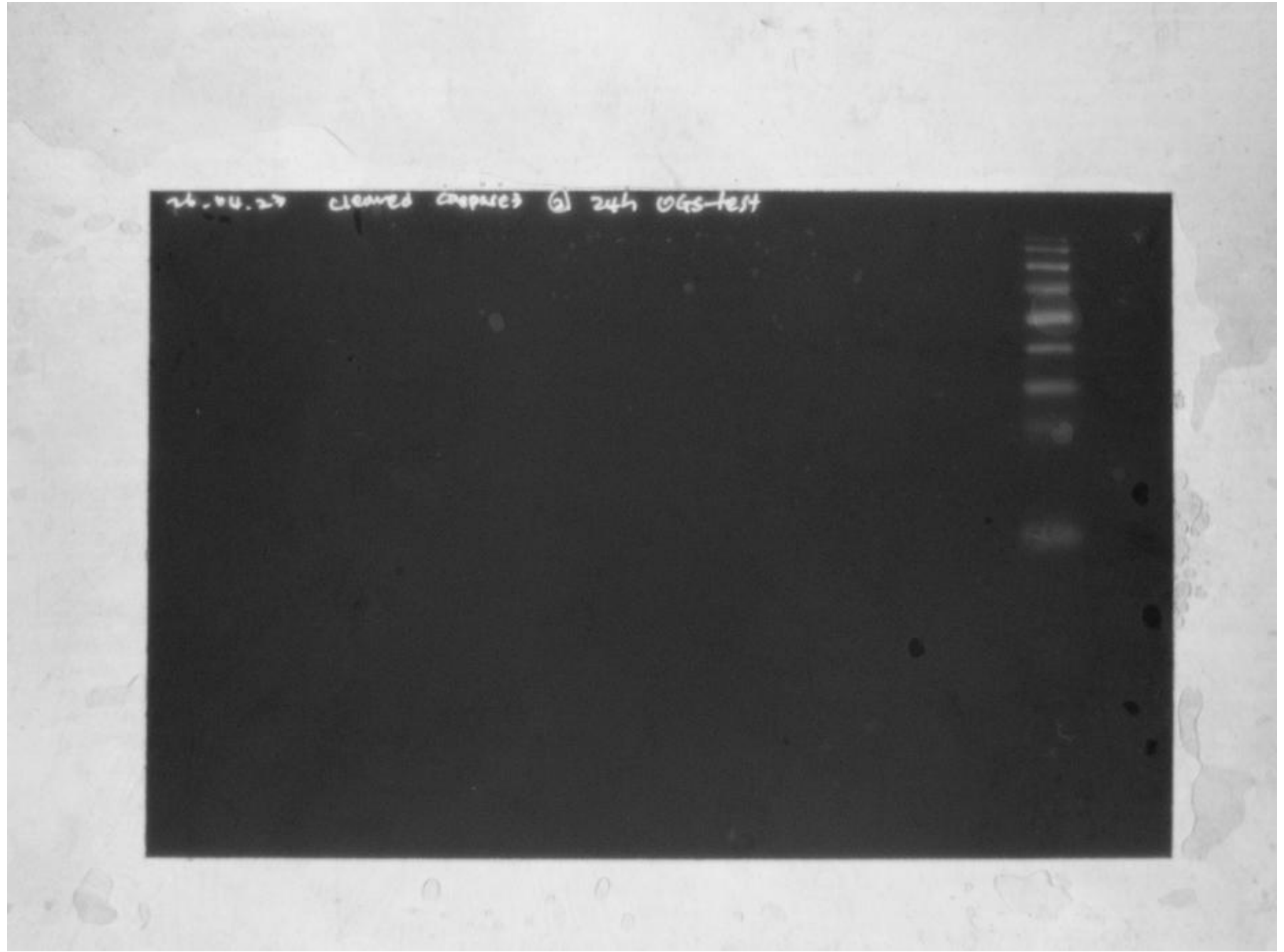

Full blot for Figure 2I

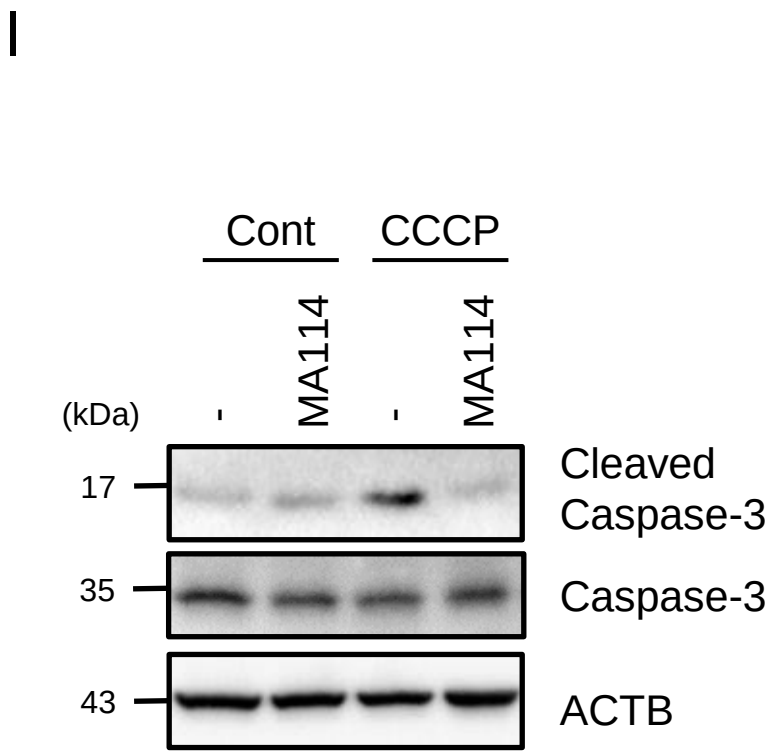

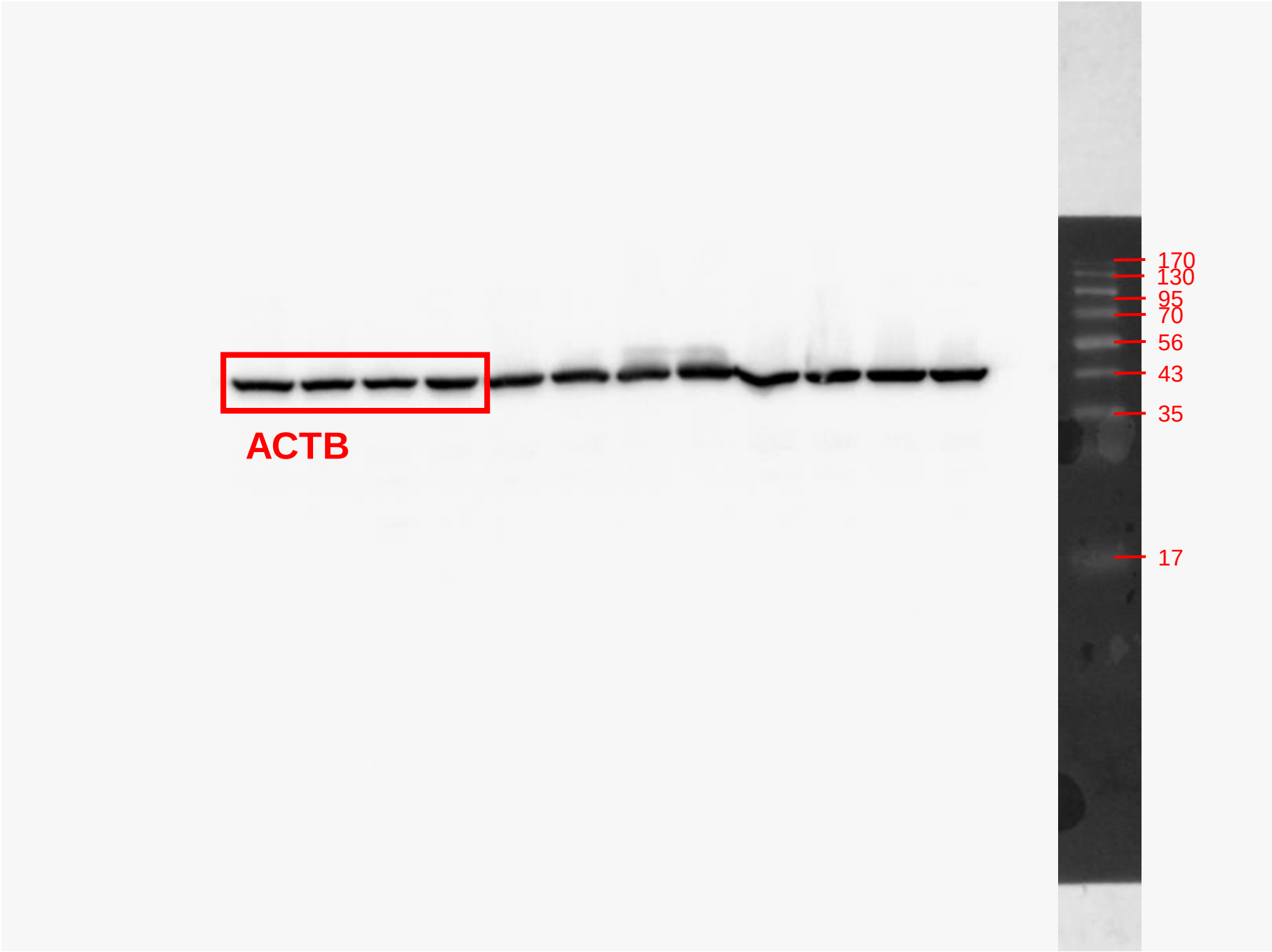

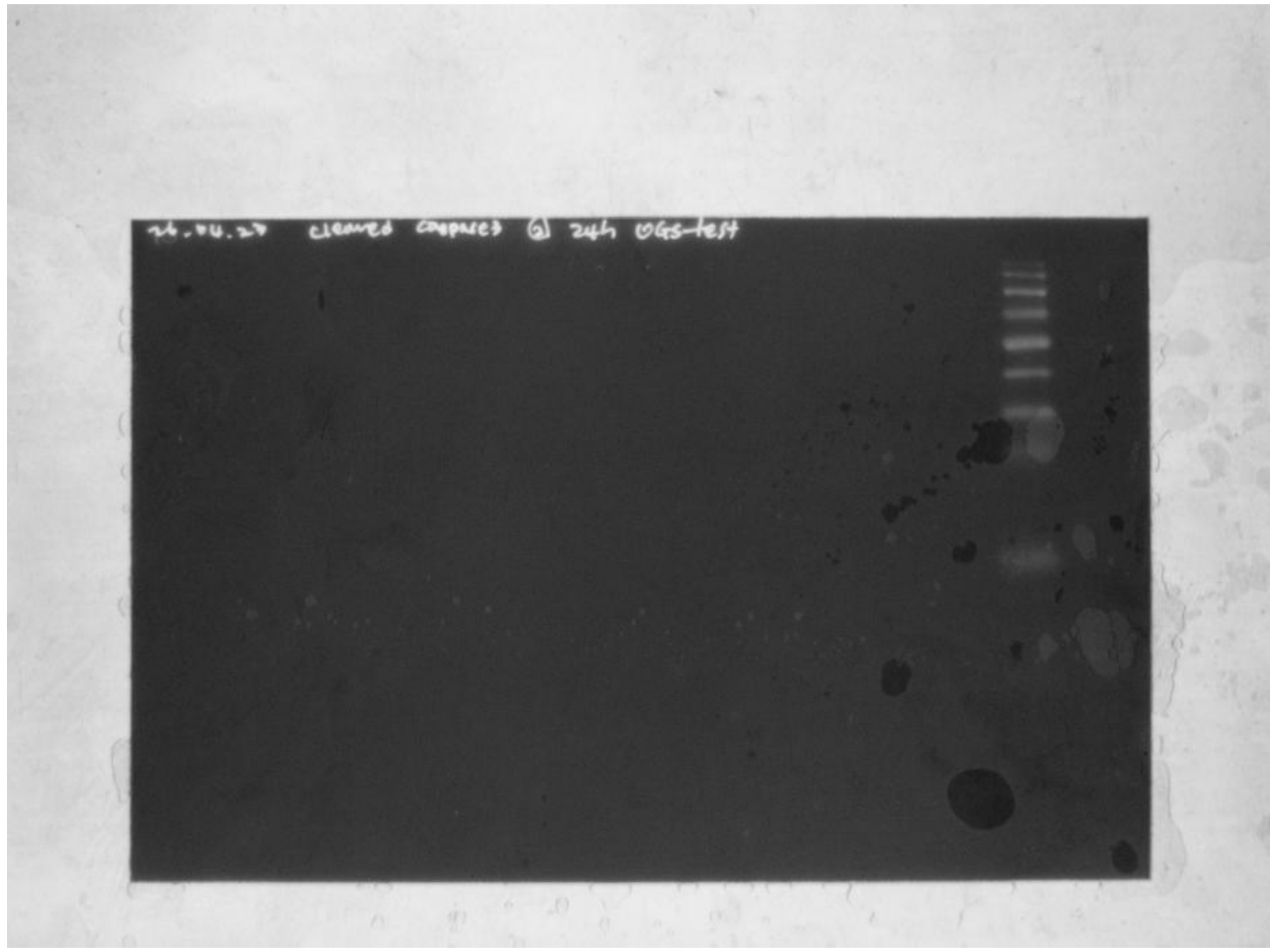

Full blot for Figure 3A

A

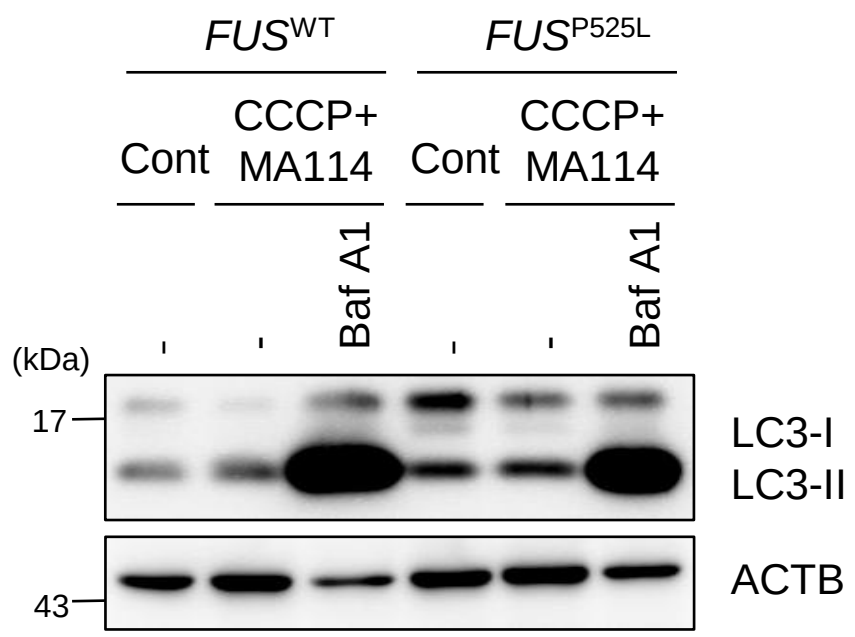

LC3

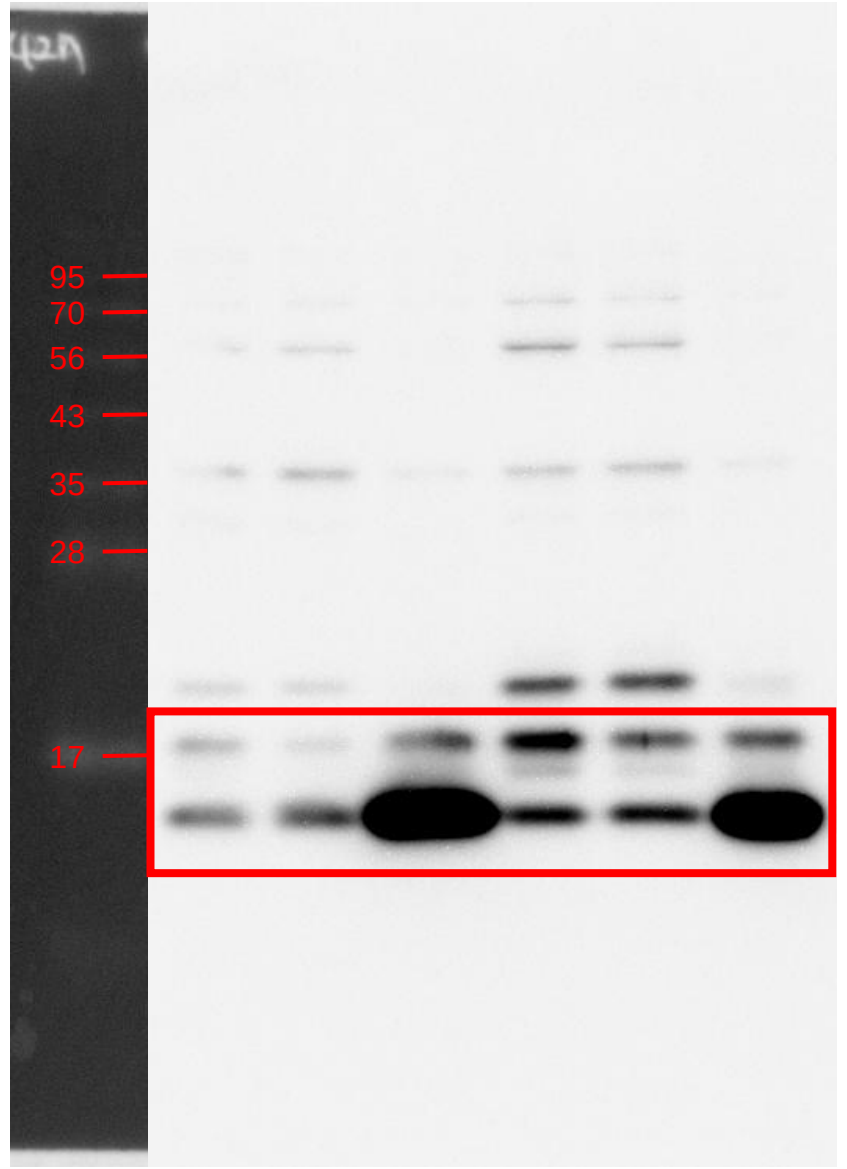

ACTB

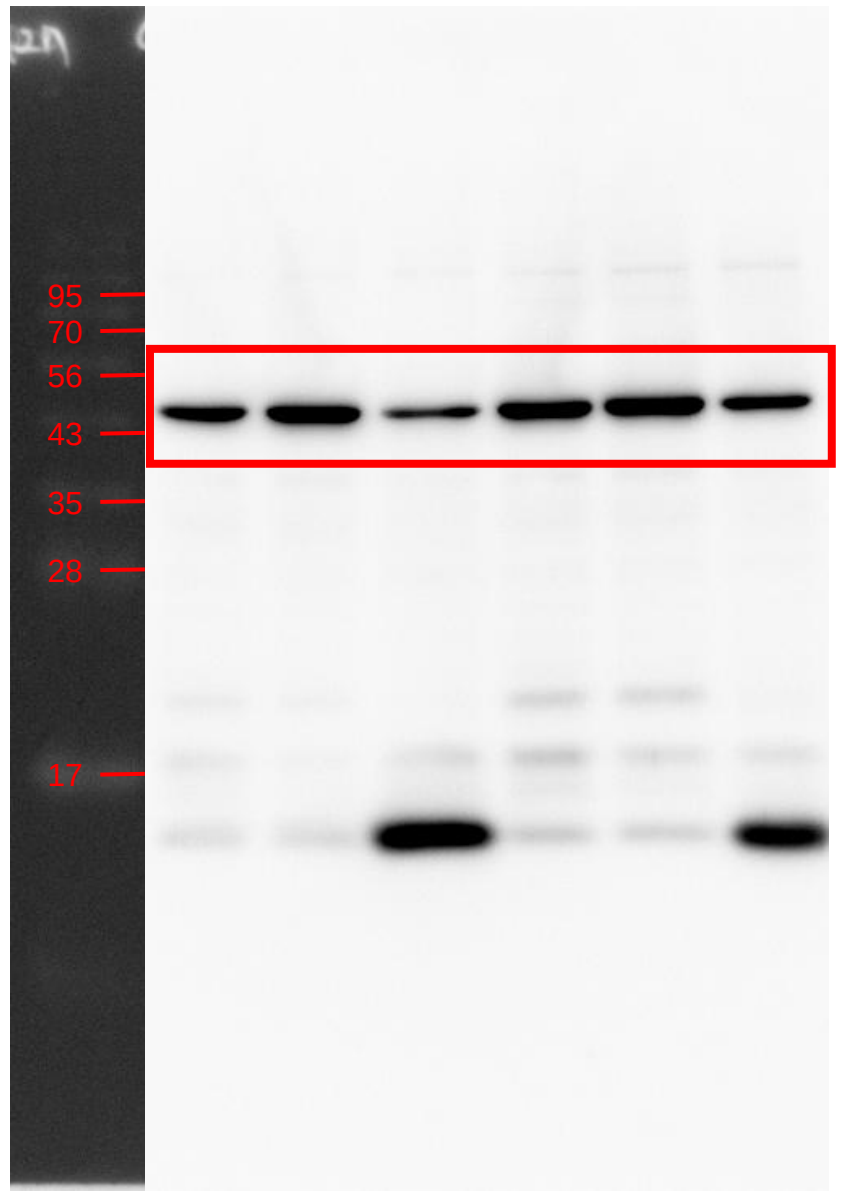

Full blot for Figure 3C

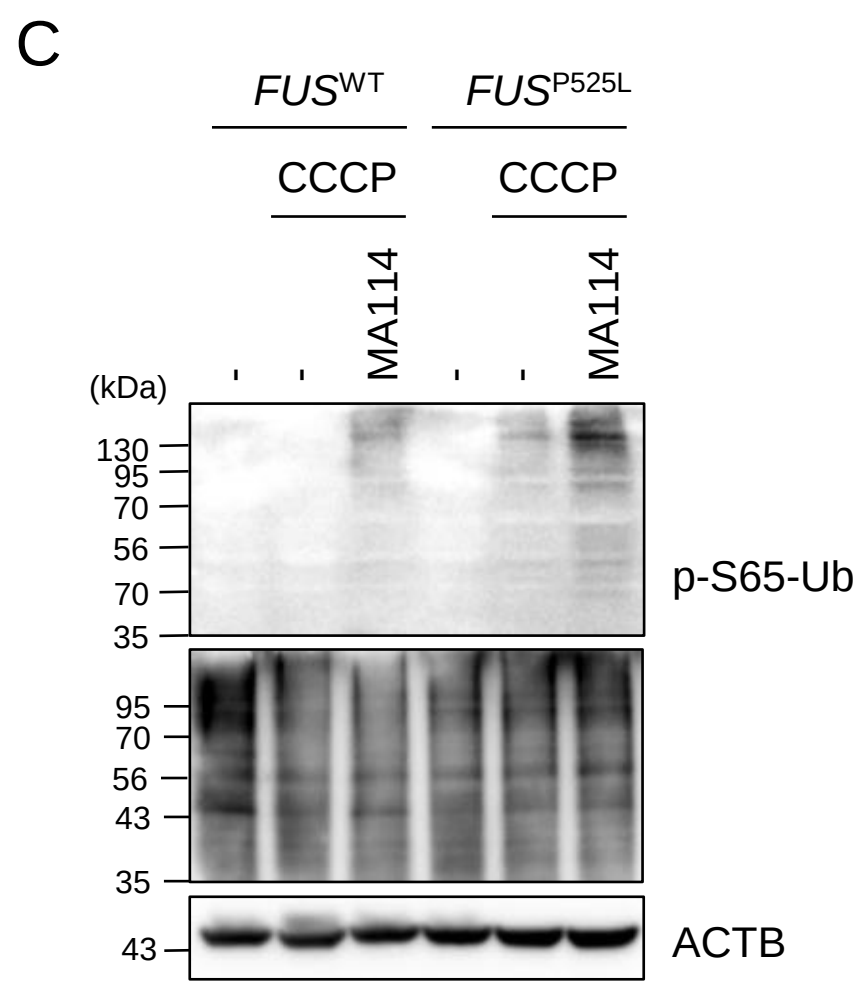

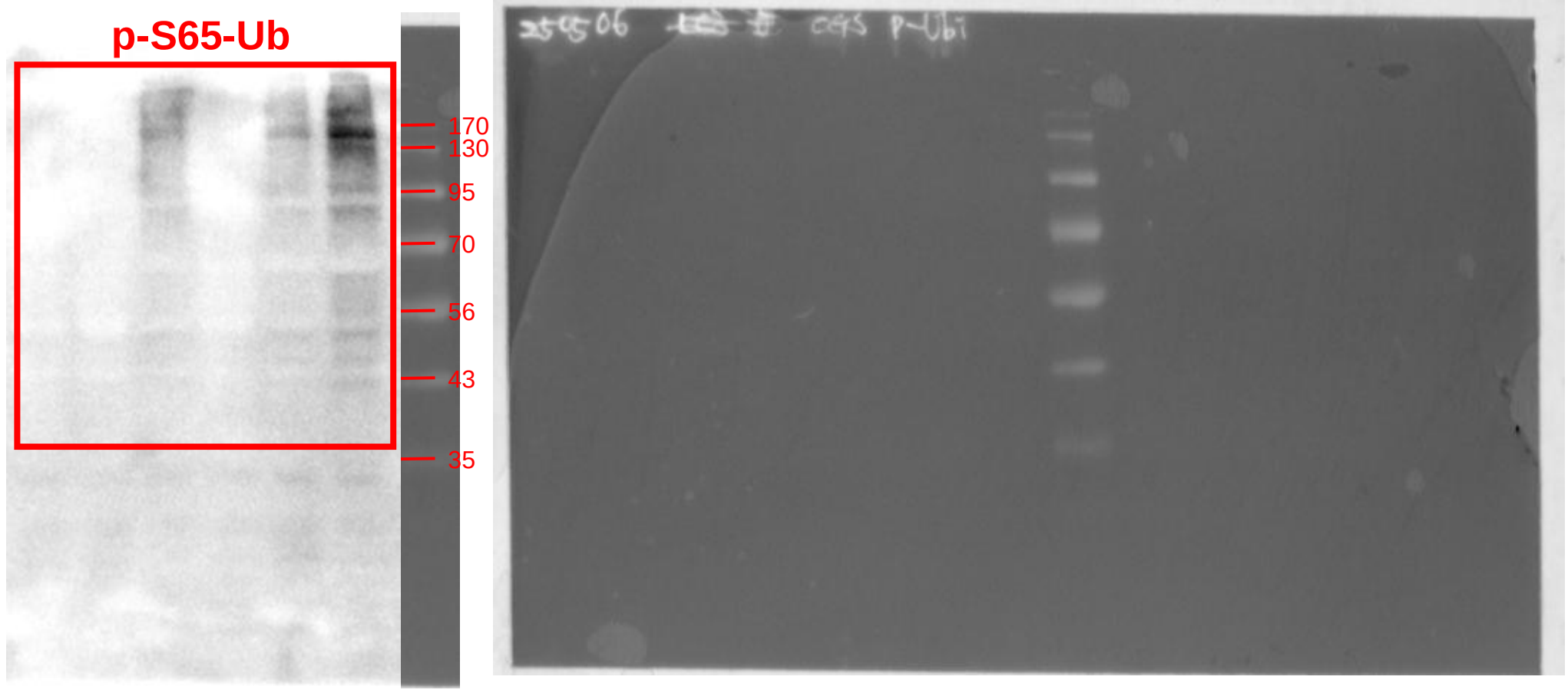

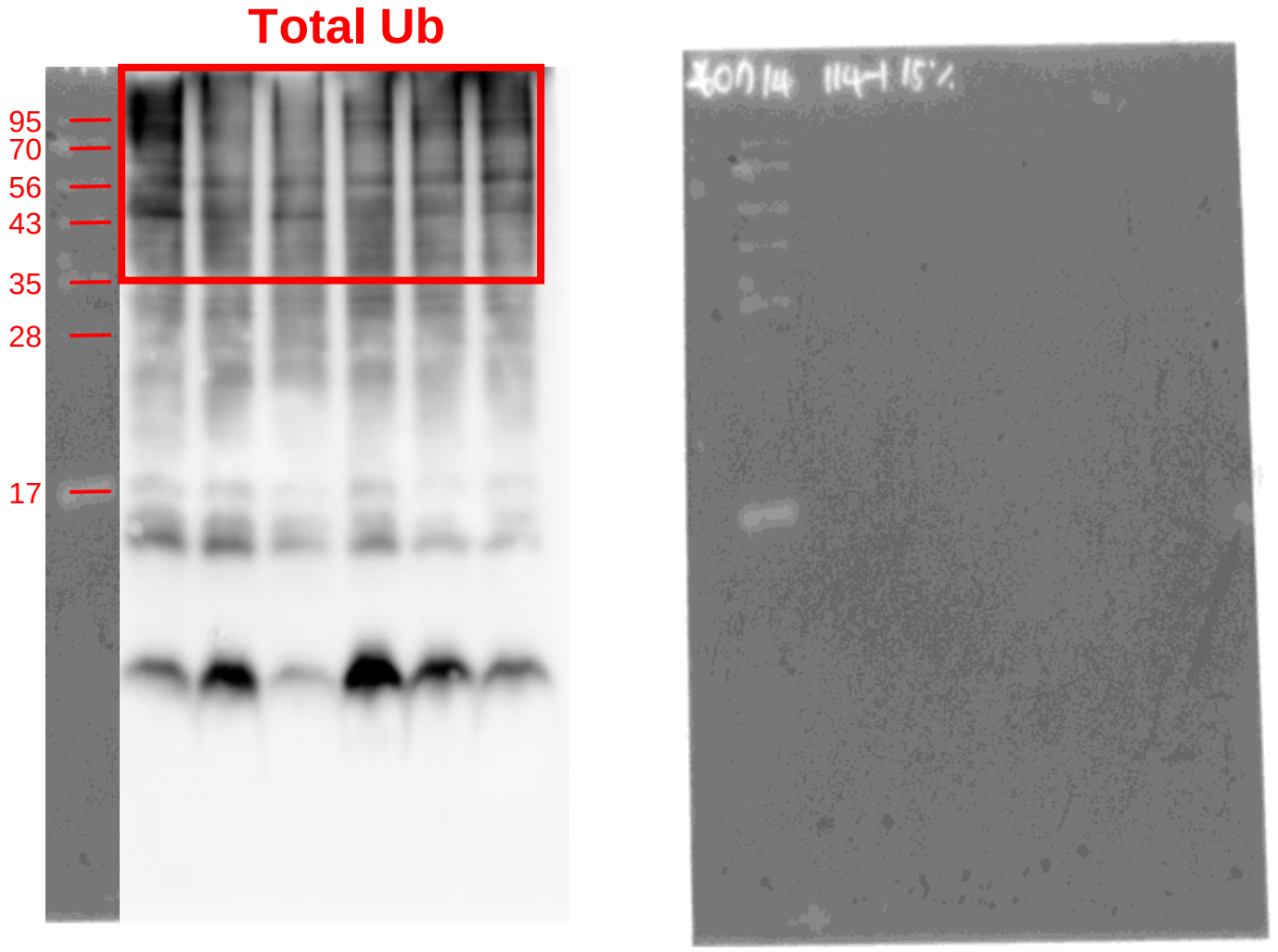

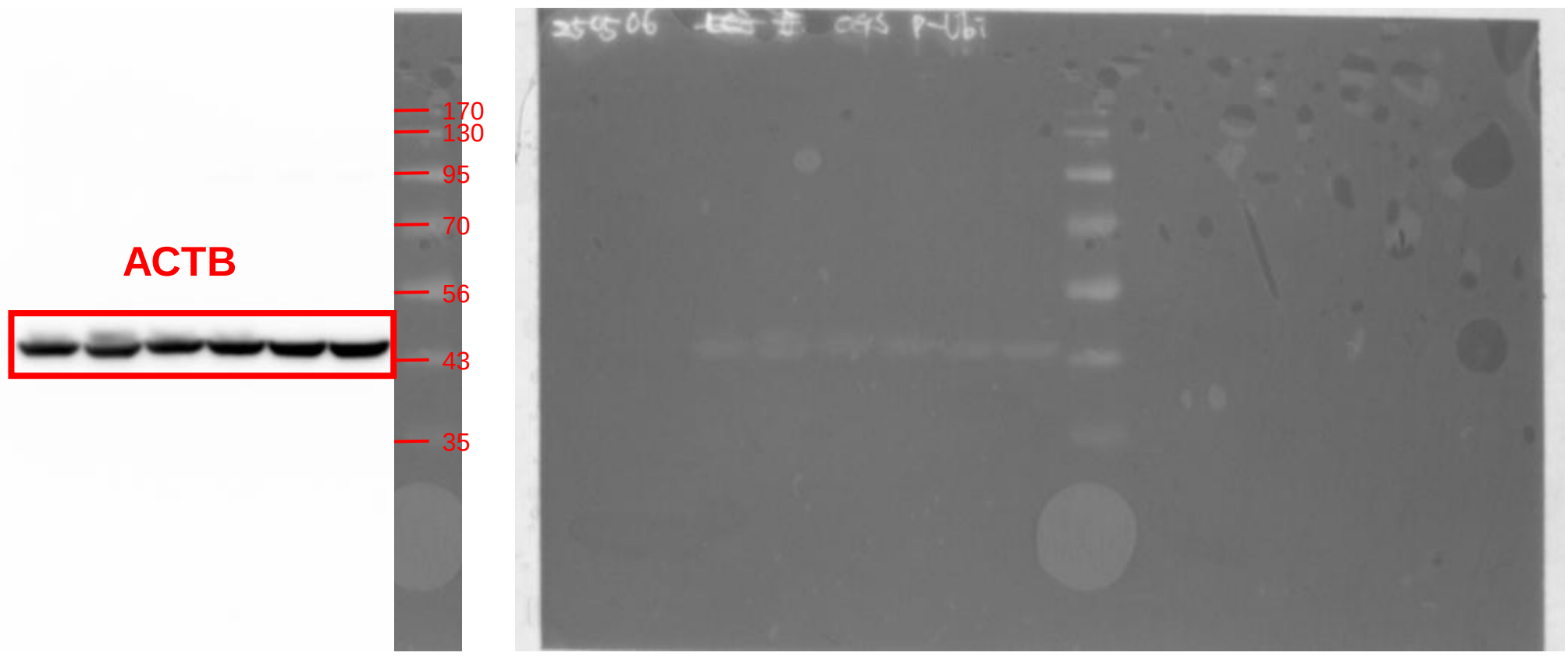

Full blot for Figure 3D

D

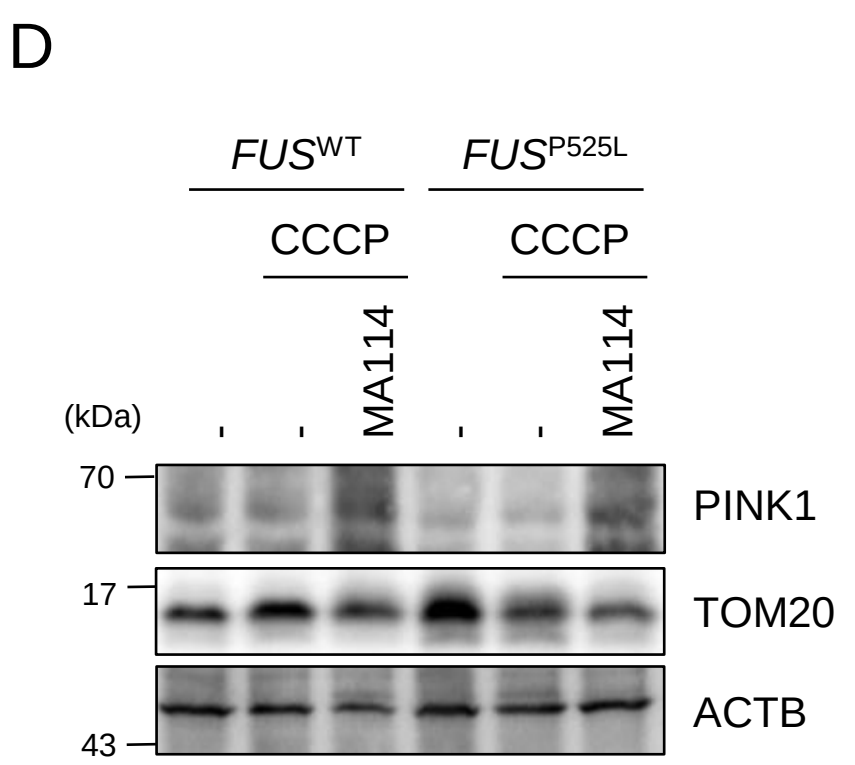

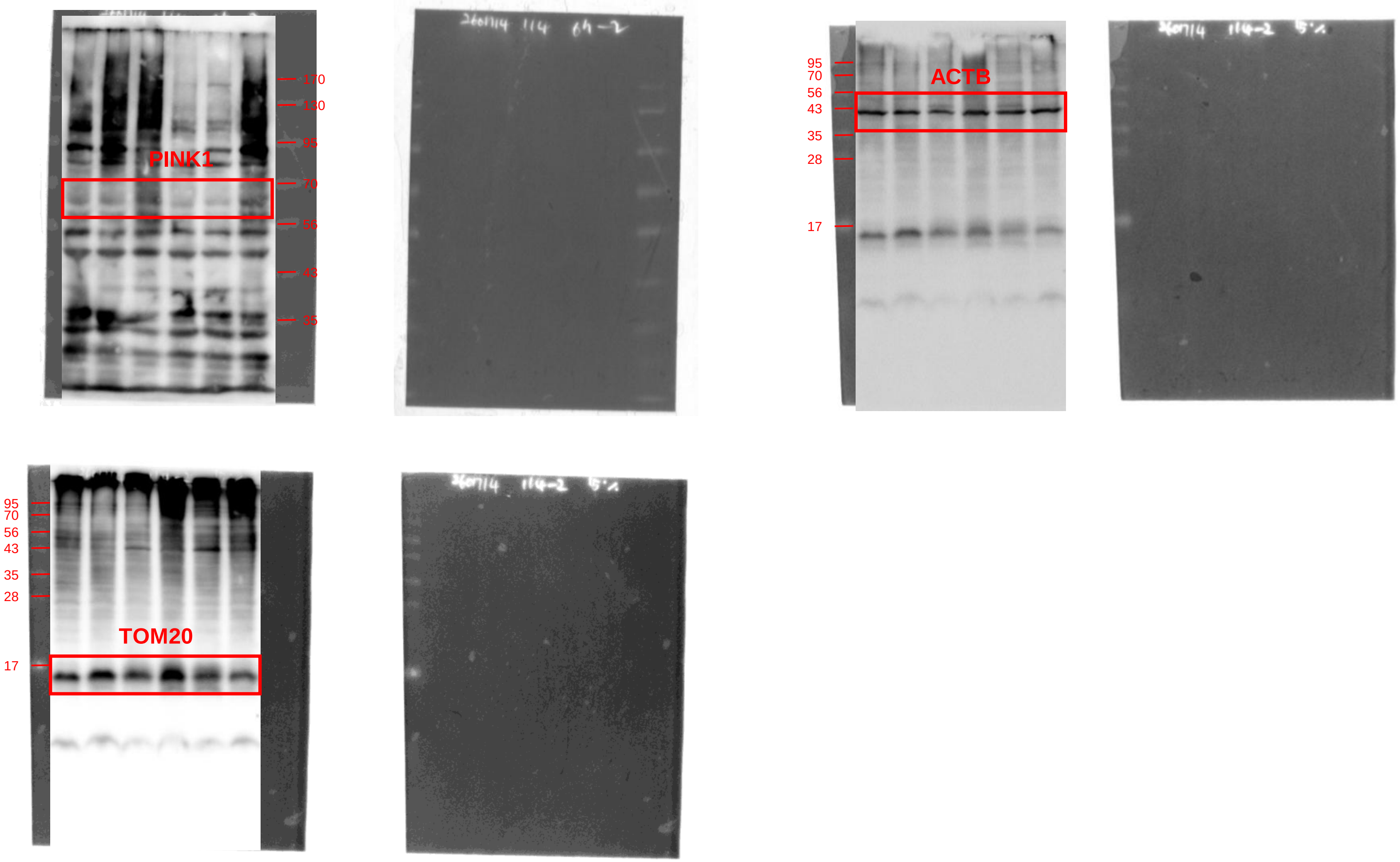

Full blot for Figure 3G

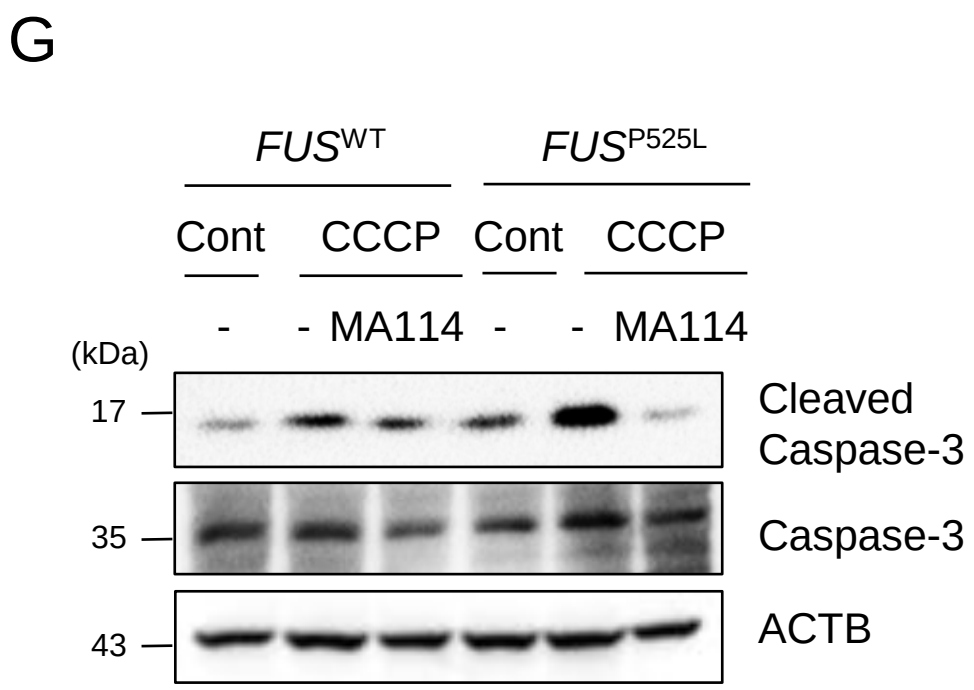

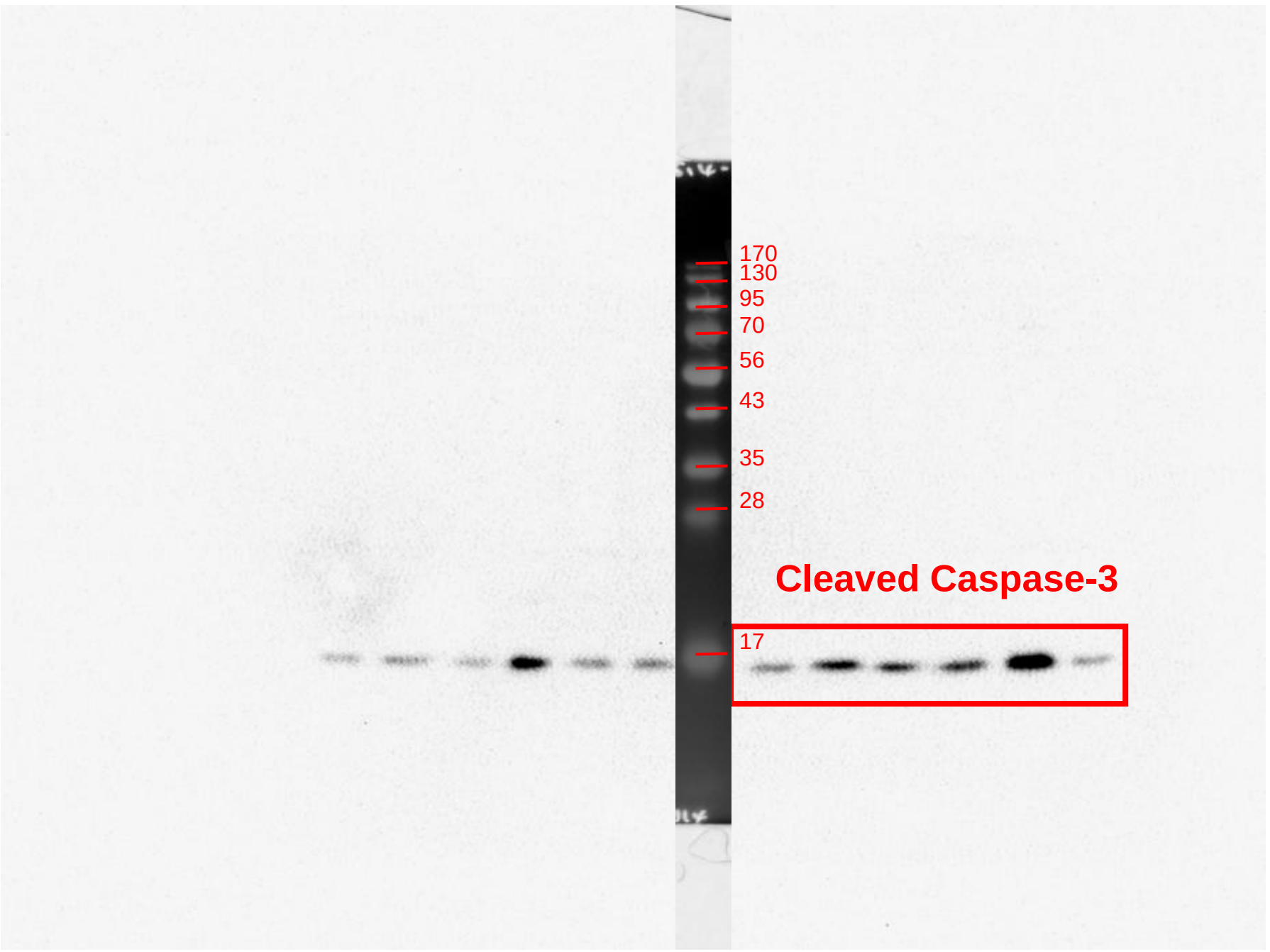

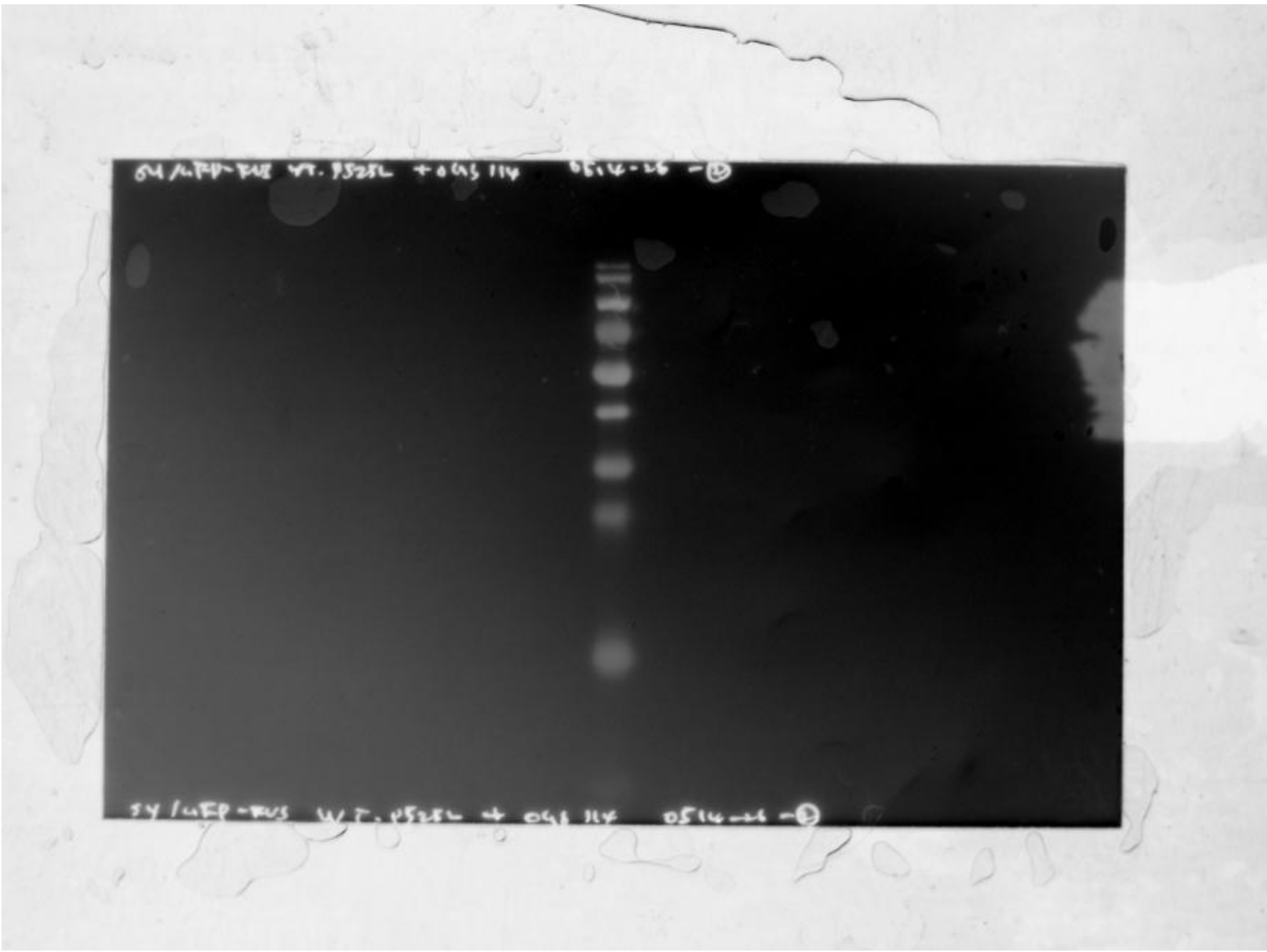

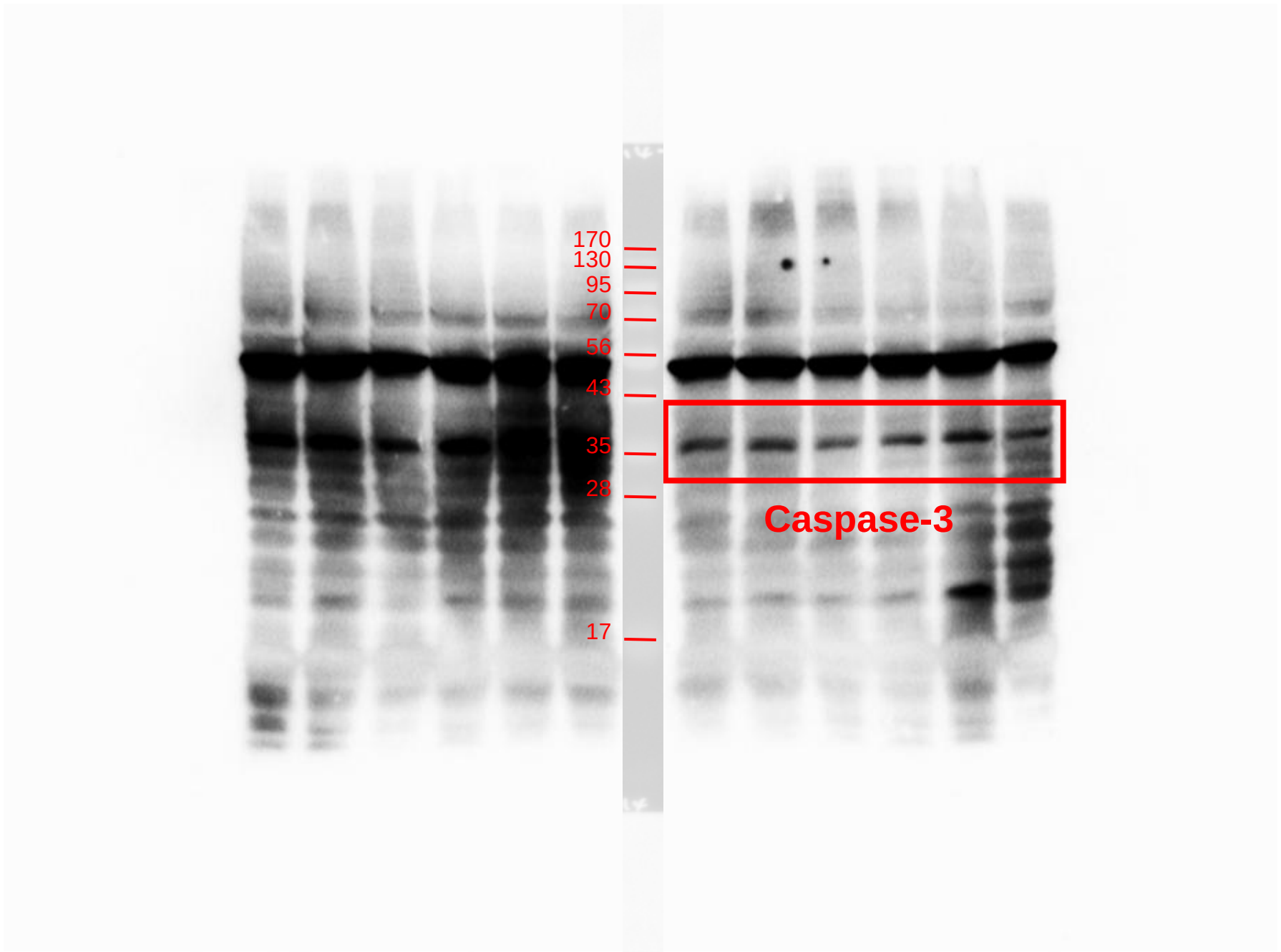

Full blot for Figure 3G

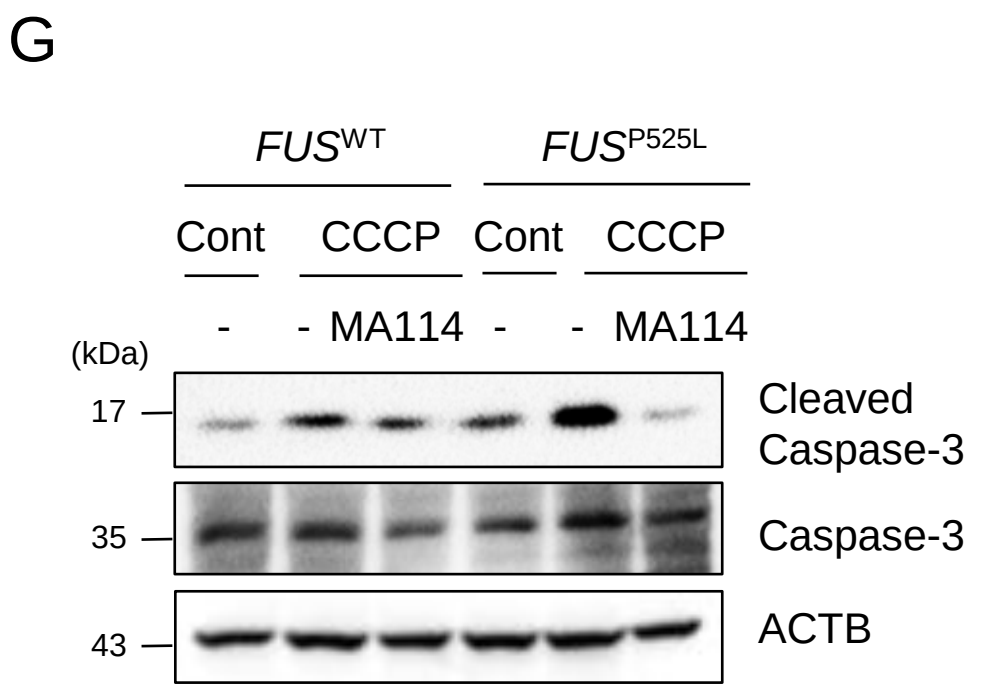

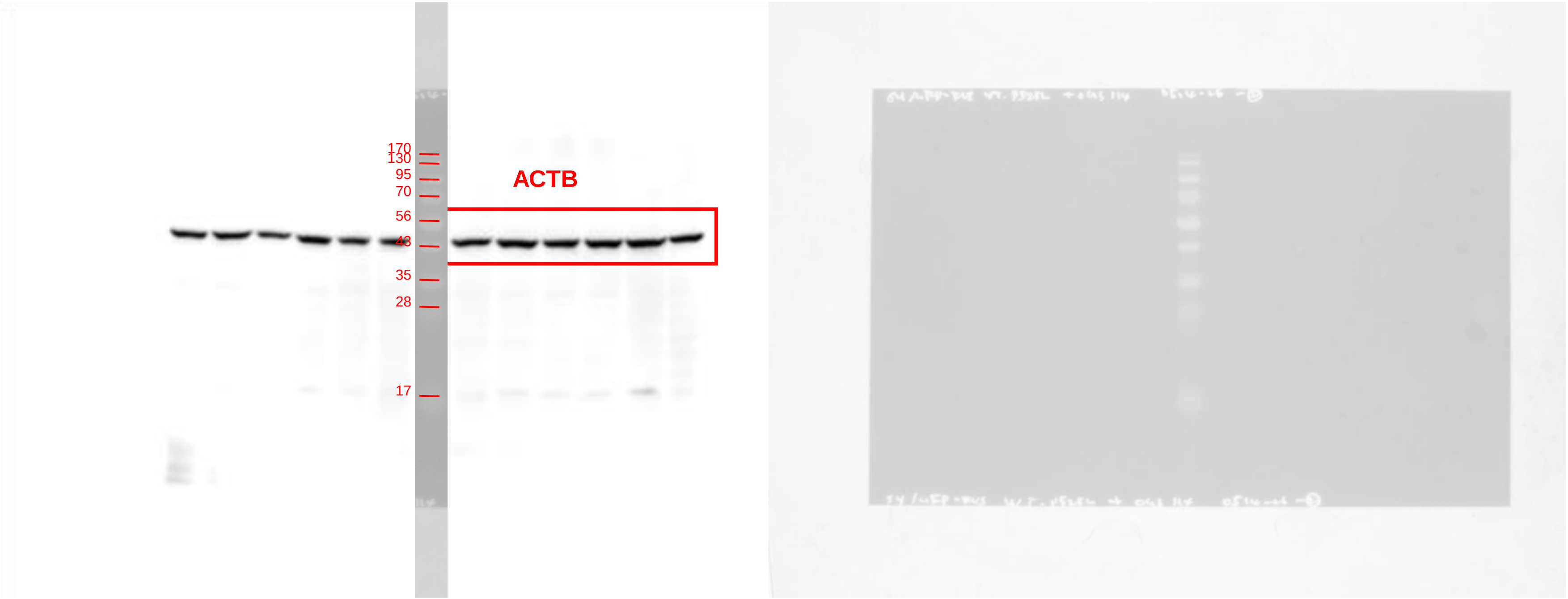

Supplement: Supplementary file 1 [file TACS_A_2736980_SM1566.pdf]
